# In Vitro Evaluation of the Anti-Diabetic Potential of Aqueous Acetone *Helichrysum petiolare* Extract (AAHPE) with Molecular Docking Relevance in Diabetes Mellitus

**DOI:** 10.3390/molecules27010155

**Published:** 2021-12-28

**Authors:** Kolajo Adedamola Akinyede, Habeebat Adekilekun Oyewusi, Gail Denise Hughes, Okobi Eko Ekpo, Oluwafemi Omoniyi Oguntibeju

**Affiliations:** 1 Department of Medical Bioscience, University of the Western Cape, Bellville, Cape Town 7530, South Africa; ghughes@uwc.ac.za (G.D.H.); okobi.ekpo@gmail.com (O.E.E.); 2 Biochemistry Unit, Department of Science Technology, The Federal Polytechnic P.M.B.5351, Ado Ekiti 360231, Ekiti State, Nigeria; habbyfat@gmail.com; 3 Department of Biosciences, Faculty of Science, Universiti Teknologi Malaysia, UTM, Johor Bahru 81310, Johor, Malaysia; 4 Department of Anatomy and Cellular Biology, College of Medicine and Health Sciences, Khalifa University, Abu Dhabi P.O. Box 127788, United Arab Emirates; 5 Phytomedicine and Phytochemistry Group, Oxidative Stress Research Centre, Department of Biomedical Sciences, Faculty of Health and Wellness Sciences, Cape Peninsula University of Technology, P.O. Box 1906, Bellville 7535, South Africa

**Keywords:** glucose uptake, drug discovery and development, α-amylase and α-glucosidase inhibitors, diabetes mellitus

## Abstract

Diabetes mellitus (DM) is a chronic metabolic condition that can lead to significant complications and a high fatality rate worldwide. Efforts are ramping up to find and develop novel α-glucosidase and α-amylase inhibitors that are both effective and potentially safe. Traditional methodologies are being replaced with new techniques that are less complicated and less time demanding; yet, both the experimental and computational strategies are viable and complementary in drug discovery and development. As a result, this study was conducted to investigate the in vitro anti-diabetic potential of aqueous acetone *Helichrysum petiolare* and B.L Burtt extract (AAHPE) using a 2-NBDG, 2-(*N*-(7-Nitrobenz-2-oxa-1,3-diazol-4-yl) amino)-2-deoxy-d-glucose uptake assay. In addition, we performed molecular docking of the flavonoid constituents identified and quantified by liquid chromatography-mass spectrometry (LC-MS) from AAHPE with the potential to serve as effective and safe α-amylase and α-glucosidase inhibitors, which are important in drug discovery and development. The results showed that AAHPE is a potential inhibitor of both α-amylase and α-glucosidase, with IC_50_ values of 46.50 ± 6.17 (µg/mL) and 37.81 ± 5.15 (µg/mL), respectively. This is demonstrated by a significant increase in the glucose uptake activity percentage in a concentration-dependent manner compared to the control, with the highest AAHPE concentration of 75 µg/mL of glucose uptake activity being higher than metformin, a standard anti-diabetic drug, in the insulin-resistant HepG2 cell line. The molecular docking results displayed that the constituents strongly bind α-amylase and α-glucosidase while achieving better binding affinities that ranged from ΔG = −7.2 to −9.6 kcal/mol (compared with acarbose ΔG = −6.1 kcal/mol) for α-amylase, and ΔG = −7.3 to −9.0 kcal/mol (compared with acarbose ΔG = −6.3 kcal/mol) for α-glucosidase. This study revealed the potential use of the *H. petiolare* plant extract and its phytochemicals, which could be explored to develop potent and safe α-amylase and α-glucosidase inhibitors to treat postprandial glycemic levels in diabetic patients.

## 1. Introduction

Diabetes mellitus (DM) is a complex global public health condition and, after cancer and cardiovascular disease, is the third most common chronic and non-contagious disease [1]. Insulin dysfunction results from a lack of pancreatic cells to release insulin (Type 1 DM) or an inadequate insulin response (Type 2 DM), both of which are known as insulin resistance (IR). IR reduces insulin-sensitive activity and is the leading cause of Type 2 diabetes worldwide, accounting for 90–95 percent of cases, and is thought to be produced by a prolonged hyperglycemic state. The contribution of reactive oxygen species (ROS) generated by the oxidative stress (OS) induced by chronic hyperglycemia is linked to the onset and progression of diabetes and its associated complications [2,3]. Chronic hyperglycemia produces antioxidant imbalances in the body; therefore, treating DM requires exogenous antioxidants, notably flavonoids, which are bioactive components of many medicinal plants. Medicinal plants are the repository of many bioactive compounds; hence, their excellent nutritional values and biological or pharmacological activities are mainly due to flavonoids [4]. Flavonoids are important bioactive plant compounds with a wide range of nutritional and health benefits as well as biological activities, such as anti-atherosclerosis, anti-inflammatory, anti-diabetic, neuroprotective, antioxidant, anti-proliferative, antimicrobial, and hepatoprotective [5,6,7,8,9,10] activities. The biological effects of flavonoids are due to their interactions with various proteins and enzymes, including cytochrome P450, laminin receptor, phospholipase A2, α-amylase, α-glucosidase [4], and many others. Interactions between flavonoids and α-amylase and/or α-glucosidase (protein or enzyme) are specific inhibitory mechanisms of prospective anti-diabetic drugs [4,11]. Flavonoids also influence insulin production, insulin signaling, carbohydrate metabolism/digestion, and glucose absorption in insulin-sensitive tissues through a variety of intracellular signaling pathways [12,13].

Digestive enzymes α-amylase and α-glucosidase that hydrolyze starch are implicated in postprandial hyperglycemia; thus, inhibition of these enzymes reduces glucose release and absorption in the small intestine. This significant anti-diabetic effect attributed to the inhibition of α-amylase and α-glucosidase could be achieved through the biological activities of flavonoids [14,15]. Acarbose, miglitol, and voglibose are examples of synthetic medicines that block α-amylase, α-glucosidase, while biguanides, such as metformin, are used to control postprandial hyperglycemia. However, their usage is restricted or discouraged because of diarrhea, stomach pain, flatulence, and other adverse effects. As a result, novel inhibitors with improved safety and efficacy profiles to reduce postprandial glycemic levels effectively and efficiently should be discovered and developed from natural products, particularly medicinal plants. Natural medicinal plant products exhibit a greater chemical variety and engage a larger chemical space than synthetic medications. Natural product pharmacophores serve as the foundation for newly synthesized pharmaceuticals with lower hydrophobicity and higher stereochemical richness than drugs derived entirely from synthetic materials [16]. The bioactive components of natural products or medicinal plants are essential therapeutic agents in biomedical and natural product research. As a result, research into their bioactive profiling is critical. It comprises a series of steps, including plant selection, collection and identification, bioactive molecule extraction and isolation, structural elucidation, and biological and pharmacological screening [17].

The *Helichrysum* genus is an aromatic plant in the *Asteraceae* family, with roughly 500–600 species found globally, including in South Africa. Diabetes mellitus, cough, cold, wound, renal disease, skin infections, chest pain, menstruation pain, fever, and hypertension have all been treated with the *Helichrysum* genera locally in the past. The antioxidant, antibacterial, antifungal, anti-ulcerogenic, anti-tyrosinase, and anti-proliferative characteristics of the *Helichrysum* genus have been investigated in scientific studies [18,19]. Many complex bioactive chemicals, such as phloroglucinols and their derivatives, chalcones, flavonoids, α-pyrones, essential oils, and terpenoids, may be found in this massive plant genus [20,21], whereas for *Helichrysum petiolare,* phenolics, flavonoids, and anthocyanins are the dominant phytochemicals [18,22,23]. A recent review indicated that some species of *Helichrysum* are under-explored for drug discovery and development [18] in many human diseases or disorders, such as diabetes mellitus. Previous reports have affirmed that a few species, such as *H. nudifolium* L. Less, *H. odoratissimum* L. Sweet, and *H. petiolare* H and B.L have traditionally been used to treat DM [24]. *Helichrysum plicatum ssp. plicatum* and *Helichrysum graveolens* have also been scientifically investigated for the treatment of DM [25,26] in drug discovery and development as an effective anti-diabetic agent. *Helichrysum petiolare* H and B.L is commonly called the silverbush everlasting plant and is called kooigoed in the Afrikaans dialect of South Africa. *Helichrysum petiolare* is a shrub with silver-grey hair covering its aromatic round-shaped leaves with whitish-cream flowers. Our previous study has profiled the antioxidant activities, total phenol, total flavonoids, and fatty acid composition of the aqueous acetone of *Helichrysum petiolare* extract (AAHPE) [in the press]. Therefore, we aimed to provide evidence for the cytotoxic screening of this extract, inhibitory activity of α-amylase and α-glucosidase, glucose uptake activity, and LC-MS analysis of flavonoids of this extract. The molecule docking of the bioactive flavonoid’s interactions for possible drug discovery and development of safe and effective anti-diabetic agents from *Helichrysum petiolare* H and B.L. was investigated. This research ultimately aims at providing and ascertaining the possible use of *Helichrysum petiolare* H and B.L extract and its phytochemicals as an alternative source of anti-diabetic agents in the treatment of postprandial hyperglycemia in type 2 diabetes mellitus.

## 2. Material and Methods

### 2.1. Chemicals

All chemicals used in this study, including metformin, 2NBDG, 2-(*N*-(7-Nitrobenz-2-oxa-1,3-diazol-4-yl) amino)-2-deoxy-d-glucose, acetone, DMSO, α-amylase and α-glucosidase enzymes, 3,5-dinitro salicylic acid, sodium hydroxide, sodium potassium tartrate, and sodium chloride, were high-grade quality of minimum of 95% and were purchased from Sigma Aldrich (St. Louis, MO, USA) and Merck (Darmstadt, Germany). The cell culture media and reagents have been indicated in the manuscript in the cell line and culture condition section.

### 2.2. Collection of Plant Material

*Helichrysum petiolare* with accession number UFH-2020-10-01 was collected from Cape Peninsula University (CPUT), Bellville, in October 2020, Western Cape, South Africa and identified by Prof. Christopher N. Cupido of the Department of Botany, University of Fort Hare, Alice, South Africa.

### 2.3. Plant Extraction

As previously reported by Nas et al. (2019), the leaves of the plants were cleaned and air-dried to a constant weight. The dried plant sample was pulverized using an electronic blender, and the ground plant was weighed. The powdered plant materials were soaked in 90% aqueous acetone in conical flasks, subjected to intermittent stirring, and warmed in the water bath at 60 °C for 2 h [27]. The mixture was filtered through Whatman cellulose filter paper under pressure using a pump. The plant material was subjected to a second extraction by soaking overnight, and the filtrates were pooled together before being subjected to a rotary evaporator. The residue or extract obtained was allowed to dry in the fume cupboard. The residual extracts were stored at −20 °C until required for use. A percentage yield of 6.3% was obtained after 97.6 g of plant material underwent the extraction process to give 6.155 g of the extract.

### 2.4. Determination of the Enzymatic Inhibitory Activity of AAHPE

#### 2.4.1. α-Amylase Inhibitory Assay

The inhibition of α- glucosidase by plant extract as described by Ali et al. (2006) using a spectrometric method [28]. In their study, the same concentration range (10–250 µg/mL) was used for the plant extract and acarbose. Here, different concentrations of plant extracts in DMSO were mixed with 4.8 mL of distilled water and 1.2 mL of 0.5% *w*/*v* soluble potato starch in 20 mM phosphate buffer (pH 6.9) containing 6.7 mM sodium chloride in test tubes. At 0 min, 600 µL of enzyme solution was added (4 units/ml in distilled water); after 3 min, 600 µL of the mixture was transferred into another test tube containing 300 µL of DNSA (1 g of 3,5-dinitrosalicylic acid (96 mM), 30 g of sodium potassium tartrate, and 20 mL of 2N sodium hydroxide to a final volume of 100 mL in distilled water, and transferred to a hot water bath maintained at 85–90 °C for 15 min. The reaction mixture in each test tube was diluted with 2.7 mL distilled water and an absorbance measurement (Thermo spectronic spectrophotometer model-biomate 3, Madison, WI, USA) was performed at 540 nm. For concentration, the blank incubation was prepared by replacing the enzyme solution with 600 µL of distilled water at the start of the reaction. The control incubations, representing 100% enzyme activity, were conducted in the same way by replacing the plant extract with 120 µL DMSO. All the tests were in triplicate. Net absorbance (A) due to maltose generated was calculated as Equation (1):

A450 nm plant extract = A450 nm Test − A450 nm blank (1)

Thus, from the value obtained, the percentage (*w*/*v*) of maltose generated was calculated from the equation obtained from the maltose standard calibration curve (0–0.1% *w*/*v* maltose). The level of the inhibition (%) was calculated as Equation (2):

%inhibition = 100 − %reaction (at t = 3 min)(2)

where, %reaction = mean maltose in sample × 100/Mean maltose in control

#### 2.4.2. α-Glucosidase Inhibitory Assay

The inhibition of α-glucosidase by plant extract is described with slight modification from [29]. Different concentrations of the plant extract (10–250 µg/mL) were incubated with 0.5 mg of the protein equivalent of crude α-glucosidase enzyme before reaction initiation with 45 mM sucrose as substrate, in a final reaction mixture of 1 mL of 0.1 M phosphate buffer (pH 7.2). The reaction mixture was incubated for 30 min at 37 °C. A total of 1000 µL of Tris base was used to stop the reaction, and α-glucosidase was monitored using the released glucose with glucose oxidase method by absorbance at the wavelength of 450 nm. α-glucosidase inhibitory activity was expressed as percentage inhibition. The enzyme inhibition data were expressed as IC_50_ values, depicting the plant extract concentration that inhibits 50% of α-glucosidase activity.

### 2.5. Liquid Chromatography-Mass Spectrometry (LC-MS) Analysis of AAHPE

The LCMS analysis was performed using the method of Standers et al., with slight modification [30]. The UPLC-MS analysis was performed with a Waters Synapt G2 quadrupole time-of-flight (QTOF) mass spectrometer (MS) connected to a water Acquity ultra-performance liquid chromatography (UPLC) system (Waters, Milford, MA, USA). Electrospray ionization was used in negative mode with a cone voltage of 15 V, desolvation temperature of 275 °C, desolvation gas at 650 L/h, and the rest of the MS settings optimized for the best resolution and sensitivity. Data were acquired by scanning from 150 to 1500 *m*/*z* in both resolution and MSE mode. Two channels of MS data were obtained in MSE mode, first at low collision energy (4 V) and the second using a collision energy ramp (40–1000V) to obtain the fragmentation data. Leucine enkephalin was used as a locked mass (reference mass) for accurate mass determination, and the instrument was calibrated with sodium formate. Separations were achieved on a 150 mm HSST3 column. An injection volume of 3 µL was used as a mobile phase consisting of 0.1% formic acid (solvent A) and acetonitrile with 0.1% formic acid as solvent B. The gradient started at 100% solvent A for 1 min and changed to 28% B over 22 min linearly. It changed to 40% B over 50 s and a wash step of 1.5 min at 100% B was achieved after re-equilibration to the initial condition for 4 min. The flow rate was 0.3 mL/min and a column temperature of 55 °C was maintained. Compounds were quantified relatively against a calibration curve established by injecting a range of catechin standards from 0.5 to 100 mg/L catechin.

### 2.6. In Vitro Studies

#### 2.6.1. Cell Line and Culture Condition

The human hepatocarcinoma cell line HepG2, ATCC^®^ HB-8065 ™, was obtained from the American Type Culture Collection (ATTC, Manassa, VA, USA). The cell line was cultured in Dulbecco’s Modified Eagles Medium (DMEM) 4.5 g/L glucose supplemented with 10% foetal bovine serum (Gibco, Life Technologies Corporation, Paisley, UK), 1% 100 U/mL penicillin, and 100 μg/mL of streptomycin (Lonza Group Ltd., Verviers, Belgium) in sterile 60 mm Petri dishes placed in a 95% O_2_ and 5% CO_2_ sterile incubator condition for proper growth. At 70–80% cell confluence, the cells were passaged using 0.1% trypsin EDTA (Lonza Group Ltd., Verviers, Belgium).

#### 2.6.2. Cell Cytotoxicity by MTT Assay of AAHPE

In a cytotoxic assay, MTT(3-(4,5-Dimethylthiazol-2-yl)-2,5-diphenyl tetrazolium bromide) mechanisms involve the ability of viable cells to reduce the MTT reagent to formazan using the nicotinamide adenine dinucleotide phosphate (NADPH)-dependent oxidoreductase enzyme. The colour change from the yellow MTT salt solution to purple formazan occurs in the mitochondria of viable or living cells. In this assay, powdered MTT at a concentration of 5 mg/mL in PBS was used. HepG2 cells with optimum established cells of 5000 cell/well of 96-well plates were seeded in 100 µL of complete DMEM culture medium for 24 h to attach. After attachment, the cells were treated by replacing the culture medium with 25 µg/mL, 50 µg/mL, 75 µg/mL, and 100 µg/mL of AAHPE concentrations while cells without plant extract served as the control for the 48 h duration. After treatment, 10 µL of MTT solution was added to every well and incubated for 4 h. The supernatant was carefully aspirated and 100 µL of DMSO was added, after which it was read at 570 nm with a BMG Labtech multi-cell plate reader. The cell viability was expressed as a fraction of viable cells relative to the control culture.

#### 2.6.3. NBDG Glucose Uptake Assay

The insulin-resistant HepG2 cell model and glucose uptake were established according to Liu et al.’s [29] method with slight modifications. Briefly, HepG2 cells were cultured in a black 96-well culture plate; after reaching confluence, the cells were treated with 10^−6^ mol/l insulin for 24 h to induce insulin resistance. The IR HepG2 cells were treated with 25 µg/mL, 50 µg/mL, and 75 µg/mL concentrations of plant extracts and 4 mM metformin (induces proliferation, safe dose) for 24 h, after which they were incubated with 100 nM insulin for 30 min. The glucose uptake was measured after incubation with 40 μM 2-NBDG for 30 min. The cells were washed with ice-cold PBS to stop the response, and 2-NBDG fluorescence intensity was measured on a microplate reader (BMG Labtech Omega^®^ POLARStar, Ortenberg, Germany) at 485 nm (excitation) and 528 nm (emission) wavelengths. The experiment was repeated three times.

### 2.7. In Silico Drug-Likeness Analysis and ADMET Profiling

The physicochemical, pharmacokinetic, and drug-likeness properties of the flavonoid’s compounds identified by LC-MS were determined. In silico ADME (adsorption, distribution, metabolism, and excretion) analysis was performed using the European Bioinformatics Institute (EBI) SwissADME online analyzer (hhttp://swissadme.ch/ (accessed on 25 October 2021)) and ADMETlab web server (http://admet.scbdd.com (accessed on 25 October 2021)) [31,32]. The numerological values of the flavonoid compounds and metformin, an anti-diabetic drug, were interpreted by the qualitative units based on the ADMETlab server explanation. The canonical SMILES for structures of all the compounds and metformin were retrieved from the National Center for Biotechnology Information (NCBI) PubChem database (https://pubchem.ncbi.nlm.nih.gov/ (accessed on 25 October 2021)) prior to the analysis.

### 2.8. Molecular Docking

#### 2.8.1. Protein Preparation

The crystal structures of α-amylase (PDB ID: 2QV4) and α-glucosidase (PDB ID: 3WEL) proteins were retrieved in .pdb format from the Protein Data Bank (PDB) (https://www.rcsb.org/ (accessed on 25 October 2021)). The PDB is a worldwide archive used to access the 3D structures of biological macromolecules (Burley et al., 2021). In this study, we adopted a single chain of α-amylase and α-glucosidase for docking analysis. MGLTools software was used for protein preparation. The water molecules and co-crystallized ligands were deleted from the macromolecule and polar hydrogens were added.

#### 2.8.2. Ligand Preparation

The initial 3D structures of the selected ligands were retrieved in .sdf format from PubChem (https://pubchem.ncbi.nlm.nih.gov/ (accessed on 25 October 2021)) and ChemSpider (http://www.chemspider.com/ (accessed on 25 October 2021)).Pub Chem and ChemSpider are publicly accessible repositories for chemical substances and their related biological activities [33,34]. The optimized structures were then converted into .pdb format using open BABEL.

#### 2.8.3. Docking Protocol

The PDB files of both ligands and proteins were converted in an extended PDB format, termed PDBQT, to perform molecular docking analysis using AutoDock 1.5.6 and AutoDock Vina. The docking protocol was used as previously reported by many studies [35,36]. The “Grid” of AutoDock 1.5.6 was used for calculating the grid parameters, and all the data regarding target proteins, ligand, grid size, and geometry were saved in the “TXT” file. Docking was performed with the grid box size set to 60 × 68 × 56 and 84 × 76 × 50 xyz points for α-amylase and α-glucosidase, respectively, with a grid spacing of 1 Å and the grid center designated at dimensions (x, y, and z): 17,453, 61,696, and 14,571 and 13,999, 16,094, and 28,018 for α-amylase and α-glucosidase, respectively. The output PDBQT files were written into a config. (configuration) file. The conformation with the lowest binding energy was considered the most stable conformation of the ligand regarding the bioactive compounds. The results were analyzed using the free version of Biova Discovery Studio 2020 client (Dassault Systèmes BIOVIA, Discovery Studio Modeling Environment, Release 2017, San Diego: Dassault Systèmes, 2016).

#### 2.8.4. Docking Method

The reference ligands were docked in the binding site of the target proteins and compared with those of the co-crystallized ligands of the target proteins (the PDB ligand of 2QV4 and 3WEL was the sulphate ion) to determine the accuracy of the docking protocol. The prepared ligand molecules were docked in the binding site of the refined α-amylase and α-glucosidase models utilizing AutoDock Vina and scored using the scoring function. The protein–ligand interactions were analyzed further for the docked poses of the ligands in the binding sites of the target proteins. The best pose was selected for further analysis of the binding interactions (including H-bond and hydrophobic interactions) of the ligands using PyMOL (The PyMOL Molecular Graphics System, version 2.2.0, Schrodinger, New York, NY, USA, 2018) and Biova Discovery Studio 2020 client (Dassault Systèmes BIOVIA, Discovery Studio Modeling Environment, Release 2017, San Diego: Dassault Systèmes, 2016).

### 2.9. Statistical Analysis

GraphPad Prism7 software was used for statistical analysis and data were expressed as mean ± SD. The significant difference was set at (*p* < 0.05) and Duncan’s test was performed.

## 3. Results

**Figure 1:**
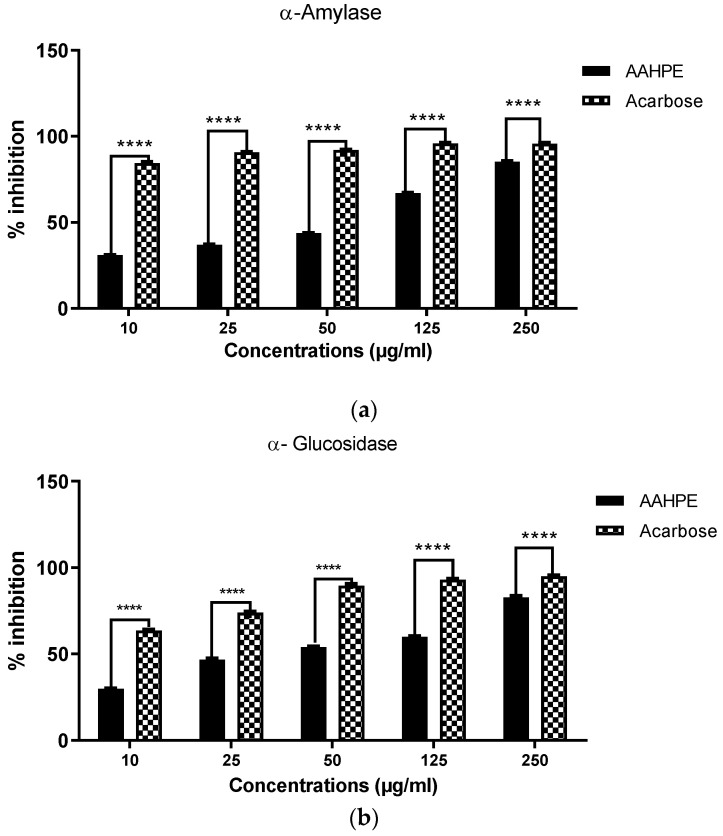
(**a**) α-amylase and (**b**) α-glucosidase inhibitory activity of AAHPE, using acarbose as a positive control (10–250 µg/mL). Values are expressed as the mean ± SD (*n* = 3), **** *p* < 0.0001 compared with the control.

The results of the α-amylase inhibition assay of AAHPE indicated concentration-dependent inhibitory action, with the highest concentration having the highest inhibition revealed in Figure 1. The IC_50_ of the extract (46.50 ± 6.17 µg/mL) was lower than that for standard acarbose (IC_50_ = 0.32 ± 0.16 µg/mL) as shown in Table 1. In the same vein, the α-glucosidase inhibitory assay of the plant extract also showed concentration-dependent inhibitory potential revealed in Figure 1. The IC_50_ of the plant extract (37.81 ± 5.15 µg/mL) was lower than that of the standard acarbose (IC_50_ = 5.38 ± 2.76 µg/mL) as shown in Table 1. At the highest concentration of 250 µg/mL, we obtained inhibitory activity of 85.25% and 82.77%, respectively, for α-amylase and α-glucosidase. Overall, the plant extract exhibits inhibition action against α-amylase and α-glucosidase and is potentially an anti-diabetic agent, especially in postprandial hyperglycemic conditions.

### 3.1. Screening of the Flavonoid’s Compound of AAHPE Using LC-MS Analysis

**Figure 2:**
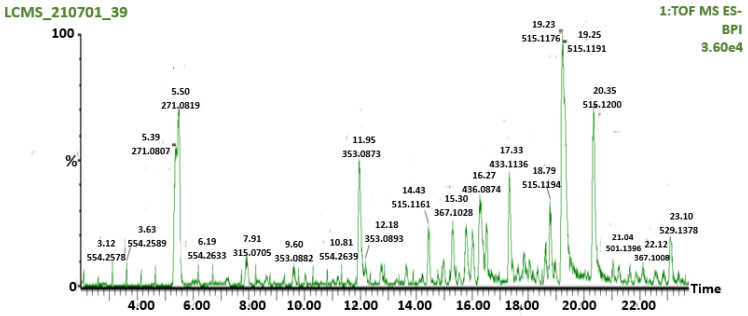
LC-MS chromatogram screening of AAHPE.

We identified 38 compounds with average R_t_ (min) and average *m/z* values and concentrations through LC-MS/MS analysis (Supplementary Materials Table S1). Previous studies have identified a positive relationship, or the ability of an increasing concentration of flavonoids, to inhibit α-amylase and α-glucosidase [37]. Hence, 19 different secondary metabolites (flavonoids) identified in Figure 2, LC-MS/MS analysis having concentrations above 100 mg/g and acarbose, an anti-diabetic drug, were docked in this study. The quantitative assessment was indicated in their concentrations (this can be seen in Supplementary Materials Table S1), and the 2D chemical structure of the selected 19 bioactive compounds of AAHPE were illustrated (Supplementary Materials Table S2). The quantitative assessment indicated the following predominant compounds: 3,5-dicaffeoylquinic acid (1727.3 mg/g), arubitin (1279.7 mg/g), 4,5-caffeoylquinic acid (1209.1 mg/g), 5-caffeoylquinic acid (826.7 mg/g), engeletin (747.9 mg/g), quercetin-3-galactoside (586.3 mg/g), 5-feruloyl quinic acid (411.6 mg/g), 3-*O*-caffeyl-4-*O*-methylquinic acid (407.1 mg/g), myricetin 3-galactoside (402.5 mg/g), and dicaffeoylquinic acid (388.7 mg/g), among others.

### 3.2. The Effect of ACHPE on HepG2 Cell Viability

**Figure 3:**
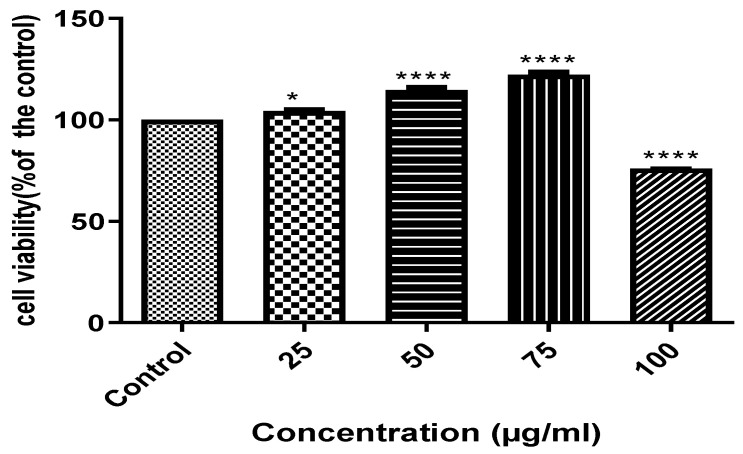
Cytotoxic screening of AAHPE at different concentrations. Values are expressed as the mean ± SD (*n* = 3), * *p* < 0.05 and **** *p* < 0.0001 compared with the control.

HepG2 cells were treated with increasing concentrations of AAHPE (25–100 µg/mL) for 48 h. The adherence to the cell viability threshold of 80% was sufficient for our work [38]. The MTT assay showed a slight increase in cell viability with the addition of the AAHPE in a dose-dependent manner between concentrations of 25 and 75 µg/mL, indicating non-toxicity compared to the control. However, at 100 µg/mL, the cell viability reduced by a quarter compared with the control. Thus, cell viability at a concentration of 100 µg/mL is around 75%, indicating slight toxicity. Hence, we adhered to concentrations of 25–75 µg/mL because the cell viability was above the threshold of 80% at these concentrations as shown in Figure 3; thus, they were regarded as very safe doses and were used in the subsequent assay in this study.

### 3.3. The Effect of AAHPE on Glucose Uptake

**Figure 4:**
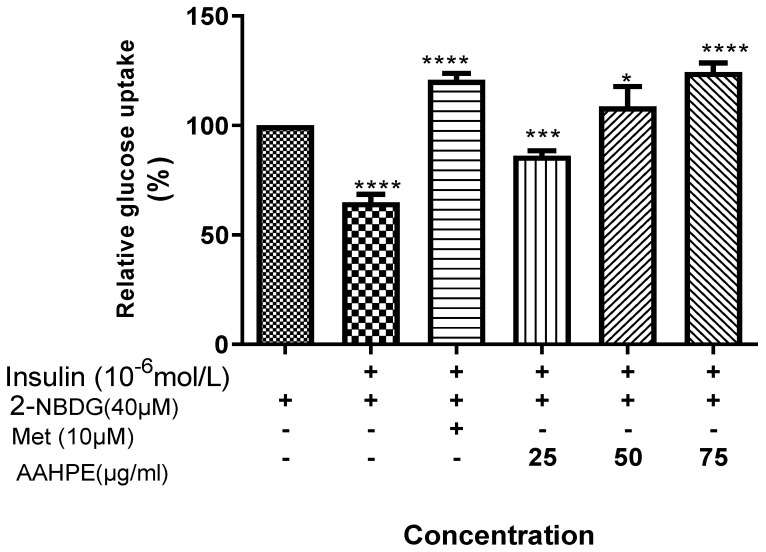
Effect of AAHPE on insulin-stimulated glucose uptake in insulin-resistant HepG2 cells. The insulin-resistant cells induced with 10^−6^ mol/L insulin were treated with AAHPE (25–75 µg/mL) concentrations or metformin for 48 h and glucose uptake was measured using fluorescent D-glucose 2-NBDG. Values are mean ± SD, * *p* < 0.05, *** *p* < 0.001 and **** *p* < 0.0001 significant compared with the control.

The glucose uptake effect in the insulin-resistant HepG2 cells indicated that the standard drug metformin increased the glucose uptake tremendously. There was increase in glucose uptake in concentration-dependent manner treated in insulin-resistant HepG2 cells with AAHPE (25–75 µg/mL). The highest concentration of the plant extract 75 µg/mL is slightly higher in glucose uptake activity than the standard metformin as revealed in Figure 4.

In this study, not all the potential compounds satisfied Lipinski’s rule of five regarding the octanol-water partition coefficient (LogP ≤ 5), molecular weight (≤500 KDa), number of H-bond donors (≤5), number of H-bond acceptors (≤10), and molecular refractivity (40–130) as tabulated in Table 2. Lipinski’s rule of five, referred to as Pfizer’s rule, is one of the techniques used to evaluate the drug-likeness of a chemical compound as it delineates the relationship between pharmacokinetics and physicochemical parameters. It determines the potential of the biological activities or pharmacological properties of a chemical compound and its effectiveness as an oral drug in humans [39,40]. We adhered to compounds with no violations or a minimum of a single violation, as violations of two or more indicate that drug candidates may not be orally active [40,41]. Hence, compounds with predicted poor oral absorption but high potency, as revealed in the Supplementary Materials, could be improved upon using an optimized absorption enhancement approach in future research.

The different ADME properties help with predictions and are essential in drug discovery and development, as shown in Table 3. A significant property is metabolism, which involves the interactions of possible drug candidates with cytochrome P450 (CYP) that ultimately lead to drug elimination through metabolic biotransformation. We observed that all the compounds did not inhibit the different CYP isoforms (CYP1A2, CYP2C19, CYP2C9, CYP2D6, and CYP3A4) except sinocrassosideA1, which is an inhibitor of CYP1A2 and CYP2C19. Essentially, CYP and P-glycoprotein (P-gp) offer protection to tissues and organisms by processing molecules or drugs synergistically. The inhibition of CYP connotes a significant cause of pharmacokinetics-related drug–drug interactions, with increased toxicity and unwanted side effects predominating because of the reduced clearance and bioaccumulation effect of the drug or metabolite [40,42]. In drug discovery and development, potential drug candidates with unfavourable absorption, distribution, metabolism, and elimination (ADME) parameters are disqualified from clinical trials [31].

### 3.4. Result of Molecular Docking

The present work revealed the binding interaction of 19 selected bioactive compounds in AAHPE and acarbose with α-amylase and α-glucosidase enzymes or protein molecules. Further, an investigation into the interaction was performed in silico using the Auto Dock Vina. Each of the 19 bioactive compounds identified with LC-MS and acarbose (standard drug, positive control) were docked, giving a clear understanding of their interaction with the active sites of both human pancreatic α-amylase (HPA) and human intestinal α-glucosidase (HIG) enzymes. As revealed in Table 4, 19 bioactive compounds present in AAHPE had a higher binding affinity for both HPA and HIG enzymes than the standard drug acarbose. The binding affinity for HPA ranged from ΔG = −7.2 to −9.6 kcal/mol compared to ΔG = −6.1 kcal/mol for acarbose, while the binding affinity for HIP enzymes ranged from ΔG = −7.3 to −9.0 kcal/mol compared to ΔG = −6.3 kcal/mol for acarbose. Arbutin (−7.0 kcal/mol) and protocatechuic acid (−6.6 kcal/mol) showed the least binding affinity for HPA and HIG enzymes, respectively, from the identified bioactive compounds but had higher binding affinity when compared with acarbose. Overall, the binding interactions of all the bioactive compounds were better than the acarbose standard anti-diabetic drug. It is important to note that the more negative the binding free energy value is, the greater the likelihood of the ligand binding to the receptor, as depicted in the interactions of the compounds and acarbose with HPA and HIG represented in Table 4. Among all compounds, sinocrassosideA1 showed the lowest binding energy to both α-amylase and α-glucosidase, whereas arbutin exhibited the highest energy in the case of both enzymes (Table 4). These results show that all selected ligands exhibit good binding affinity with our target proteins. Moreover, we selected acarbose as a standard drug, docked against both targeted proteins. The results were compared with the selected anti-diabetic bioactive compounds (Table 4), which further suggest their affinity for the targeted proteins. This depicts the high possibility of molecular interaction between these bioactive compounds and the enzymes α-amylase and α-glucosidase.

This likely denotes that those bioactive compounds can act individually or synergistically, which results in the good inhibitory activity against HPA and HIG obtained in the in vitro AAHPE inhibition assay.

**Figure 5:**
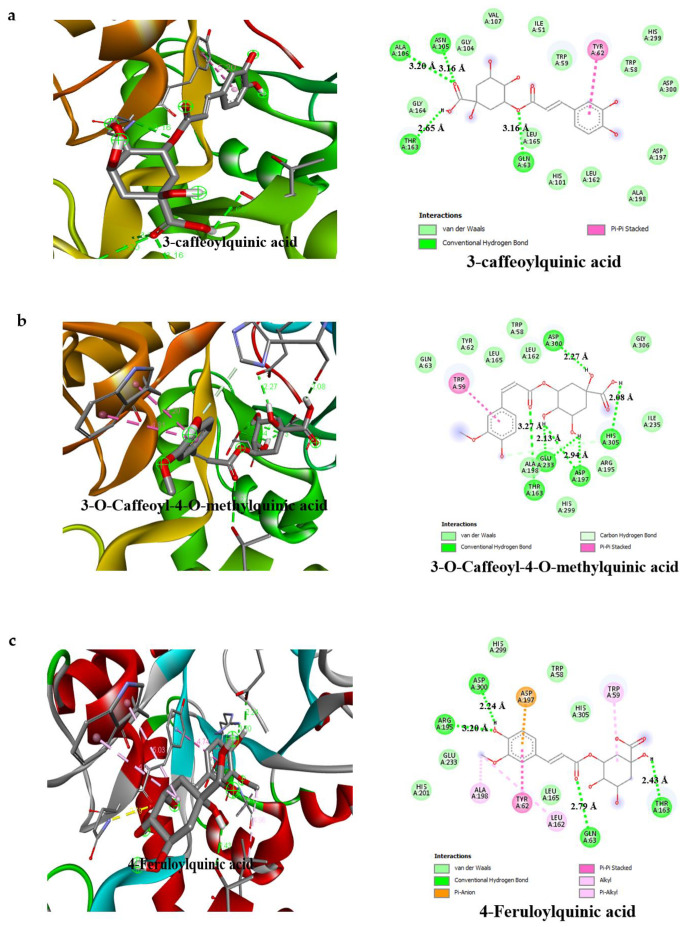

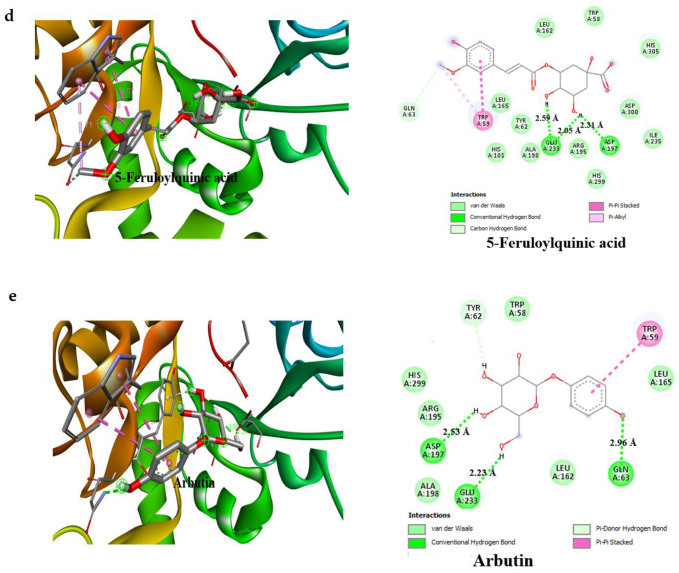

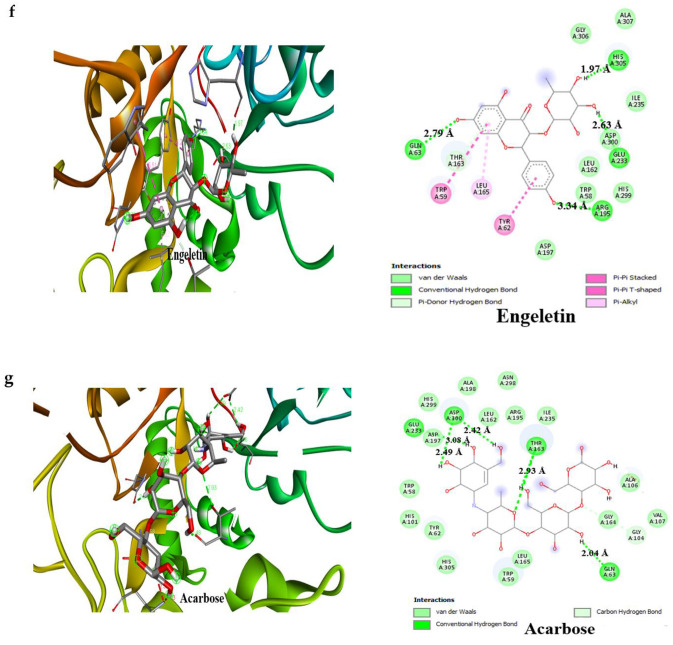

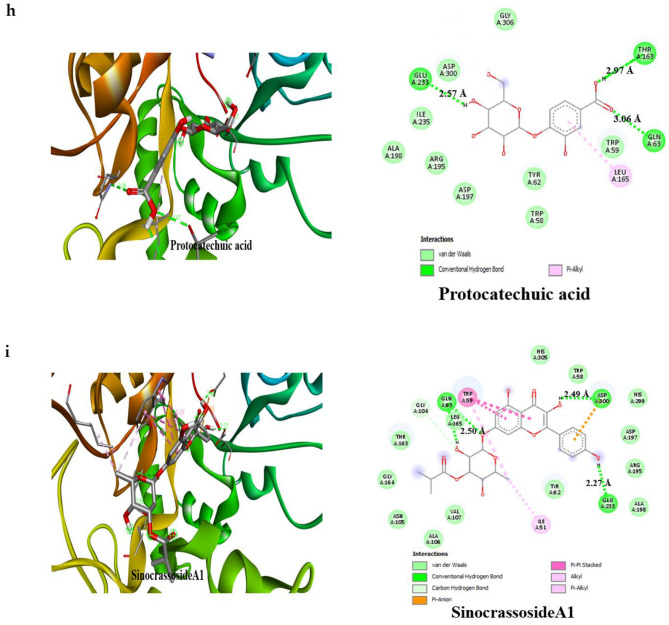
Model of the interaction and the 2D Structure of α-amylase protein with (**a**) 3-caffeoylquinic acid, (**b**) 3-*O*-Caffeoyl-4-*O*-methylquinic acid, (**c**) 4-Feruloylquinic acid, (**d**) 5-Feruloylquinic acid, (**e**) Arbutin, (**f**) Engeletin, (**g**) Acarbose, (**h**) Protocatechuic acid, and (**i**) SinocrassosideA1.

**Figure 6:**
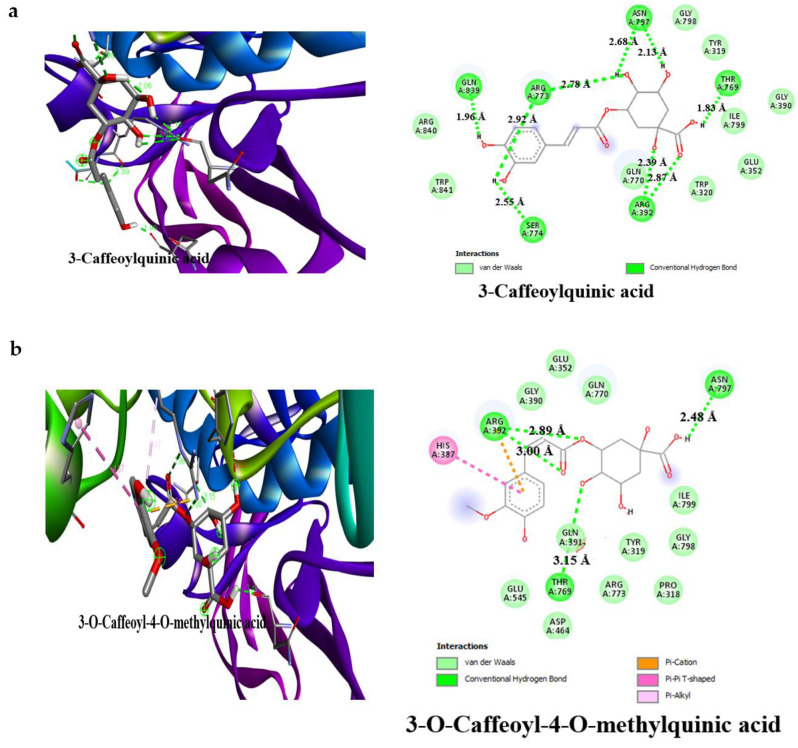

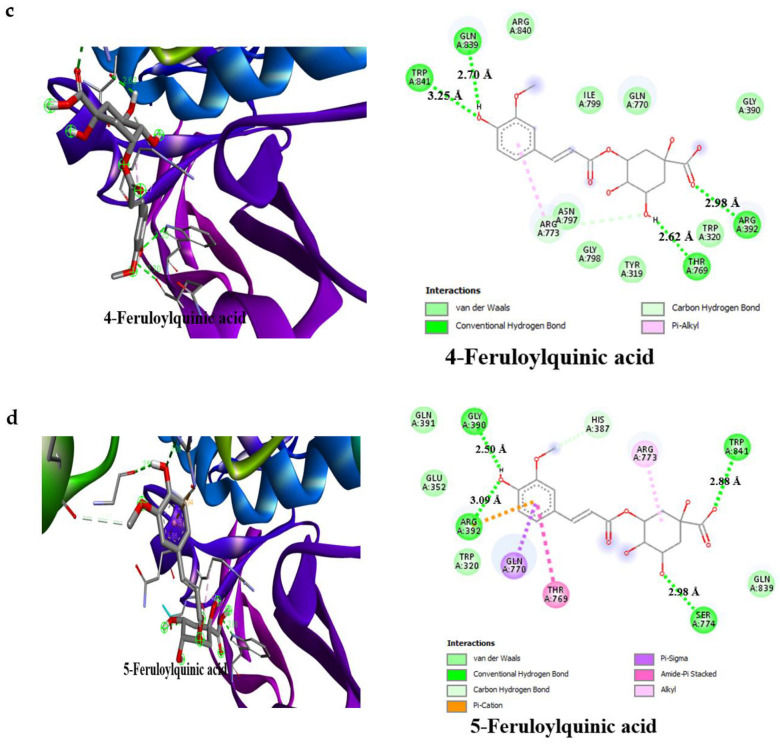

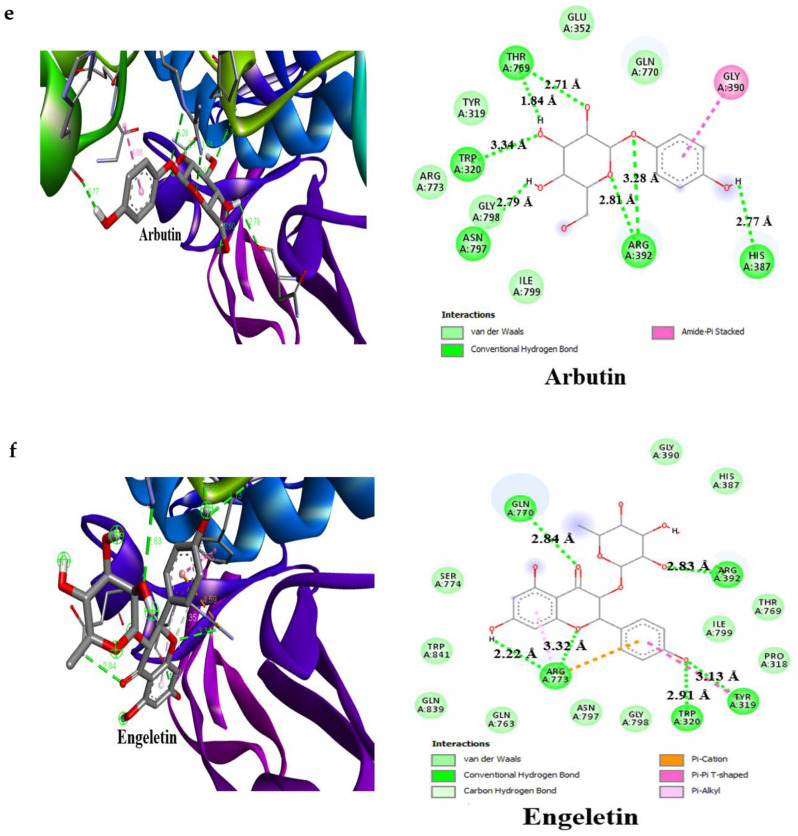

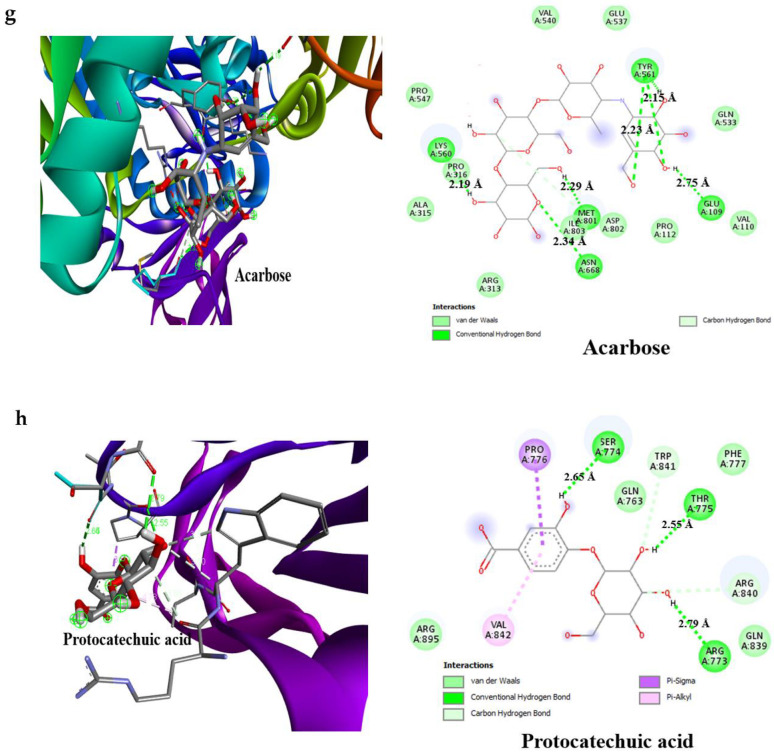

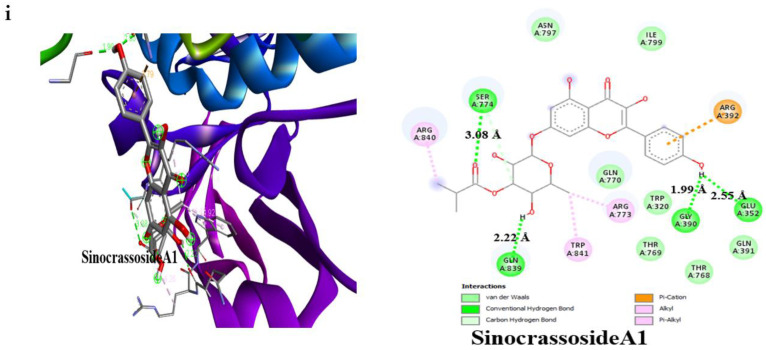
Model of the interaction and the 2D Structure of α-glucosidase protein with (**a**) 3-caffeoylquinic acid, (**b**) 3-*O*-Caffeoyl-4-*O*-methylquinic acid, (**c**) 4-Feruloylquinic acid, (**d**) 5-Feruloylquinic acid, (**e**) Arbutin, (**f**) Engeletin, (**g**) Acarbose (**h**), Protocatechuic acid, and (**i**) SinocrassosideA1.

The detailed interactions of the bioactive compounds of AAHPE with HPA and HIG enzymes was depicted by the visualization and analysis of the docking results, completed using the free version of Biova Discovery studio Visualiser 2020 software. The software revealed the best docking poses, amino acid residues in such interactions, and contributing bond types (conventional hydrogen bond, carbon–hydrogen bond, pi–sigma, pi–pi, pi–alkyl, van der Waals, and others) of the bioactive compounds with HPA and HIG enzymes. From Figure 5 and Figure 6, it was revealed that the bond interactions of the bioactive compounds of AAHPE are numerous and vary. However, van der Waals, carbon–hydrogen, and conventional hydrogen bonds are available in the acarbose interaction with HPA and HIP enzymes. Generally, weak intermolecular interactions, such as hydrophobic and van der Waals bonds, are adjudged to promote greater affinity of the ligand for the target protein with other bonds, thus stabilizing energetically favoured ligands [43]. The best ligand-binding poses in the catalytic domain of HPA and HIG after docking, with the amino acid residues involved in the interaction, are shown in Figure 5 and Figure 6, with a propensity that a set of similar important amino acid residues could be the target to facilitate improved drug efficacy. We observed a similar set of amino acid residues for HPA (Gln63, Thr163, Glu233, and Asp300) and for HIG (Glu109, Lys560, Tyr561, Asn668 and Met801) in the standard drug acarbose with inhibitory potential. However, variation was observed in the interaction of bioactive compounds of AAHPE with the main catalytic residues of Ser774, Arg733, Gln770, Arg392, Gln63, Trp59, and Thr163 for the HPA and HIG enzymes. These additional amino acid residues participating in the interaction might contribute to the inhibitory activity of these bioactive compounds. Our docking results for the bioactive compounds showed that many were actively involved in hydrogen bonding with eight polar residues, including aspartic acid, serine, histidine, threonine, glutamine, asparagine, glutamic acids, and tyrosine. Other crucial interactions, such as weak van der Waals forces, pi–sigma, and pi–pi interactions, were also found to increase the binding of bioactive compounds with protein binding pockets.

## 4. Discussion

Diabetes mellitus (DM) is a global health concern with metabolic disorders of multiple aetiologies. DM is often characterized by the perturbations of biomolecules, including carbohydrates, fats, and proteins, with defects of insulin secretions or actions, or both [44]. Reducing hepatic glucose generation, increasing insulin production and sensitivity, inhibiting gluconeogenesis, and decreasing glucose absorption are important targets employed in developing synthetic anti-diabetic agents. However, limitations and side effects, such as weight gain, gastrointestinal disorders, headache, peripheral oedema, and hypotension, associated with using these synthetic agents, the cost, and accessibility remain major hindrances [45,46]. Hydrolysis of complex starch, oligosaccharides, and disaccharides by the pancreatic α-amylase, together with glucose uptake by the intestinal α-glucosidase, have been implicated in the postprandial glucose level in type 2 diabetes [47] with its attendant complications. Essentially, the regulation of α-amylase and α-glucosidase biological functions (inhibition) is critical to the treatment regimen. This implies that new drug candidates with potent inhibition of the intestinal α-glucosidase and mild inhibition of pancreatic α-amylase and with a safety profile would be valuable to drug discovery and development for diabetes mellitus.

In the field of drug discovery and development, both experimental and computational strategies are valid and complimentary. The use of information from chemical and biological entities, such as ligands or targets, in the identification and optimization of new drugs, the elimination of compounds with undesirable characteristics (poor activity and/or poor absorption, distribution, metabolism, excretion, and toxicity (ADMET)), and ensuring that the process of drug discovery and development is streamlined are benefits of computational techniques [31,40,48]. Overall, enriched compounds with drug-likeness, lead-likeness, active qualities, and elimination of molecules with inactive, reactive, toxic, or poor ADMET/PK properties are highly desired in drug discovery and development [32,40,48]. In vitro investigation of plant extracts for anti-diabetic potential or activities is essential. The exploration of bioactive compounds present in plant extracts for anti-diabetic activities provides the basis for the discovery of bioactive compounds that are potent inhibitors of intestinal α- glucosidase and mild inhibitors of pancreatic α-amylase with better potency and safety profiles which is urgently necessary.

*Helichrysum petiolare* H and B.L, commonly called the silverbush everlasting plant, is traditionally used to treat different ailments and has been investigated scientifically to manage disease conditions [18]. Our research on *Helichrysum petiolare* involves drug discovery and development of the plant extract and its polyphenolic bioactive compounds as safe and effective potential anti-diabetic agents, considering their inhibitory activity against α-amylase and α-glucosidase enzymes. Reducing blood sugar, especially the postprandial blood sugar level, is an effective and attractive strategy in treating diabetes mellitus. Many natural products from plant sources are potential therapeutic agents; hence, fast screening of these natural products through molecular docking is important and can quickly meet this demand. Docking represents ligand and protein interactions using a computational approach for molecular recognition. It combines and screens databases for the accuracy of prognostication of protein structures and protein–ligand complexes for principal structure-based drug design [49,50,51]. Therefore, we evaluated the potential of the anti-diabetic effect of *Helichrysum petiolare* extract and its phytochemicals as α-amylase and α-glucosidase inhibitors. We observed relative safety of the AAHPE via cytotoxic screening, which is similar to other research work [52], and strong inhibitory activity when compared with acarbose. The non-cytotoxicity, good inhibition of α-amylase and α-glucosidase, and excellent glucose uptake activity of AAHPE at the highest concentration of the extract over metformin indicated anti-diabetic or hypoglycemic properties. The anti-diabetic property of *Helichrysum petiolare* is linked to repositories of the phytochemicals or bioactive compounds [12,52].

It is worth pointing out that from our study, bioactive compounds such as 3-caffeoylquinic acid, 3-*O*-caffeoyl-4-*O*-methylquinic acid, arbutin, engeletin, protocatechuic acid, and sinocrassosideA1 support Lipinski’s rule of five, while deviations from the criteria of Lipinski’s rule of five for oral bioavailability is indicated by violating more than two of Lipinski’s five rules. In view of this, the drugs formulated using these bioactive compounds that fulfilled Lipinski’s rule of five may have some significant advantages over the synthetic compound (acarbose). Interestingly, acarbose violated three out of the five rules and has been reported to be metabolically unstable [53,54].

Natural products are more complex than synthetic compounds, and drugs based on natural product structures exhibit better chemical diversity and occupy larger regions of chemical space than drugs of completely synthetic origins [16,55]. Components or compounds obtained through LC-MS analysis from *Helichrysum petiolare* extract, including protocatechuic acid, arbutin, engeletin, 3-caffeoylquinic acid, are reported to elicit anti-diabetic properties through the inhibition of α-amylase and α-glucosidase enzymes [56,57,58].

In this study, the detailed interaction of the best conformation of the docking results revealed the best docking poses of the bioactive compounds and their binding sites on α-amylase and α-glucosidase in Figure 5 and Figure 6, respectively. This involved various bonding interactions, namely conventional hydrogen, carbon–hydrogen, pi–sigma, pi–pi, pi–alkyl, etc. Based on the literature, the strength of the π–π interaction for stabilization of a structural complex is comparable to the strength of hydrogen bond at an excited state [59]. While in the ground state, the loss of the π–π interaction does not affect the active-site conformation, but results in a reduction in the rate of chemical activity approximately 20–30-fold [60]. However, hydrophobic or van der Waals interactions could promote ligand affinity for the target protein [43]. Therefore, this study evaluated the binding affinity between ligand and protein complexes by assessing binding energy, H-bonds, pi–pi interactions, and van der Waals interactions. As shown in Figure 5, the bioactive compounds in AAHPE bind with one or two reported essential binding residues, such as TRP59, ASP197, and GLU233 of α-amylase [61,62]. Aside from these essential amino acid residues, other amino acids, such as Arg195, Thr163, His305, Gln63, His299, Ala106, Asn105, and Asp300, formed hydrogen bonds, as shown in Table 4. In contrast, α-glucosidase interacts with the binding pocket of the following listed amino acid residues: Asp323, Arg392, Ser774, Thr769, His387, Arg773, Asn797, Gly390, Gly839, and Tyr841, which play an important role in phytochemical binding (Supplementary Materials Table S3).

It is noteworthy that sinocrassosideA1 had the lowest binding energy but interacted with the reported important catalytic site amino acid residues TRP59, ASP197, and GLU233 via a conventional hydrogen bond [GLU233 (2.63 Å)], pi–pi interaction (TRP59), and van der Waals interaction (ASP197) (Figure 5i). Meanwhile, sinocrassosideA1 interacted with α-glucosidase through four hydrogen bonds (Ser774, Gln839, Gly390, and Glu352) and other bonds, as shown in Figure 6i. Similar interactions were observed for all other bioactive compounds in AAHPE. Similarly, acarbose showed interactions with α-amylase ASP197 via a van der Waals interaction, TRP59 through a carbon hydrogen bond, and GLU233 via a conventional hydrogen bond (Figure 5g), while having the least binding affinity (Table 4). Interestingly, acarbose interacted with the same key amino acid residues involved in catalysis at the active site of α-amylase and α-glucosidase (Figure 5 and Figure 6). This likely implies the inhibition of α-amylase and α-glucosidase by these bioactive compounds through a similar mode of action as acarbose. The bioactive compounds in the target proteins (α-amylase and α-glucosidase) were less than 3.5 Å, suggesting resilient hydrogen bonding between protein and ligands. Our docking results for the bioactive compounds sinocrassosideA1, arbutin, engeletin, protocatechuic acid (Figure 4 and Figure 5), isorhamnetin-3-galactoside, methyl 3,5-di-*O*-caffeoylquinate, 1,4-dicaffeoylquinic acid, 3,4-dicaffeoylquinic acid, and 5-caffeoylquinic acid, among others (Supplementary Materials Figure S1 and Figure S2), were actively involved in hydrogen bonding with eight polar residues, including aspartic acid, serine, histidine, threonine, glutamine, asparagine, glutamic acids, and tyrosine. Other crucial interactions such as weak van der Waals forces, pi–sigma, and pi–pi interactions were also found to increase the binding of phytochemicals with the protein binding pockets (Figure 5 and Figure 6).

Our docking analysis suggests that most of the bioactive compounds in AAHPE could compete with the substrate for the enzyme’s active site in a similar manner as acarbose does [61,62,63]. Acarbose has been reported in the literature to be a competitive inhibitor of α-amylase and α-glucosidase [62,64,65]. This phenomenon is further confirmed by the present study’s findings, as revealed by the similar binding site occupied and binding pose assumed by acarbose (the standard inhibitor) and the bioactive compounds, as shown in Figure 5 and Figure 6. Aside from the binding pose/binding site occupied, all bioactive compounds were bound to the enzymes near the catalytic site domain where acarbose binds (Figure 5 and Figure 6). Moreover, this suggests that these bioactive compounds contribute to the overall α-amylase and α-glucosidase inhibitory effect of the AAHPE, which could be by the same competitive mode of inhibition as acarbose [65].

On the contrary, some bioactive compounds, such 3-caffeoylquinic acid, 4-feruloylquinic acid, and isorhamnetin 3-galactoside, had a higher binding affinity than acarbose towards α-amylase (Supplementary Materials Table S3). A similar result was observed for 3,5-dicaffeoylquinic acid with α-glucosidase (Supplementary Materials Table S3). The bioactive compounds did not compete with acarbose for the active site, since they were bound to the proteins at different binding sites, as shown in Figure 5a,c and Figure S2d,h (3-caffeoylquinic acid, 4-feruloylquinic acid and 3,5-dicaffeoylquinic acid, Isorhamnetin 3-galactoside). This indicates their propensity to contribute to the overall α-amylase and α-glucosidase inhibitory effect of the AAHPE via the non-competitive mode of inhibition. Interestingly, all the bioactive compounds in AAHPE investigated in this study inhibit both α-amylase and α-glucosidase, either via the competitive or non-competitive mode of inhibition. This was corroborated with the inhibition assay of AAHPE showing high inhibitory effect, which may be due to the synergetic effect of the bioactive compounds present in the extract. This study is novel and significant to the best of our knowledge as it is the first in silico study on the inhibitory activity of bioactive phytochemicals identified from AAHPE against α-amylase and α-glucosidase as a therapeutic target for the treatment of diabetes mellitus. Findings from this study indicated that the extract of AAHPE and its phytochemicals examined could be a promising therapeutic agent with better therapeutic efficacy than acarbose and could be a potential anti-diabetic agent with strong inhibitory activity against α-amylase and α-glucosidase.

## 5. Conclusions

This research reports the AAHPE chemical constituents identified using LC-MS/MS analysis as well as the remarkable in vitro anti-diabetic potential of AAHPE. Additionally, a combined physicochemical, pharmacokinetics, drug-like properties, and molecular docking study was performed for α-amylase and α-glucosidase with anti-diabetic constituents of AAHPE to assess new potential therapeutic drug candidates. LC-MS/MS analysis revealed phytochemicals or bioactive compounds, of which those with high concentrations were chosen for the molecular docking analysis. We affirmed that the phytochemicals sinocrassosideA1, engeletin, 4-feruloylquinic acid, 3-*O*-caffeoyl-4-*O*-methylquinic acid, protocatechuic acid, 3-caffeoylquinic acid, and arbutin are novel in fulfilling Lipski’s rules and are potential safe and potent α-amylase and α-glucosidase inhibitors among the array of phytochemicals in this study. The molecular docking showed hydrogen bonds and other interactions as they relate to the importance of binding energy and the stability of complexes of these phytochemicals and various amino acid residues in the active site of the two enzymes that confer them as α-amylase and α-glucosidase inhibitors. Further investigations of in vivo and clinical trials are warranted. Conclusively, we postulate that these bioactive compounds should be considered as safe and potent inhibitors of diabetes mellitus, controlling postprandial hyperglycemia.

## Figures and Tables

**Table 1:** The IC_50_ inhibitory effect of AAHPE and acarbose on the α-amylase and α-glucosidase enzymes.

IC_50_	AAHPE (µg/mL)	Acarbose (µg/mL)
α-amylase	46.50 ± 6.17	0.32 ± 0.16
α-glucosidase	37.81 ± 5.15	5.38 ± 2.76

**Table 2:** Predicted pharmacokinetic parameters (ADME properties) of the compounds selected from plant LC-MS analysis.

Compounds	LogP_o/w_ (MLOGP)	LogSw (ESOL)	MW (g/mol)	HBA	HBD	Reactivity (40–130)	tPSA	Solubility (mg/mL)	Rotatable Bonds (RoB)	*n* Violation
3-caffeoylquinic acid	0.96	−1.62	354.31	9	6	83.50	164.75	8.50	5	1
3-*O*-Caffeoyl-4-*O*-methylquinic acid	1.47	−1.84	368.34	9	5	87.97	153.75	5.38	6	0
4-Feruloyl quinic acid/5-Feruloylquinic acid	1.47	−1.84	368.34	9	5	87.97	153.75	5.38	6	0
Arbutin	1.07	−0.71	272.25	7	5	62.61	119.61	5.27	3	0
Engeletin	1.77	−3.09	434.49	10	6	103.95	166.14	3.55	3	1
Metformin	0.34	0.29	129.16	2	3	36.93	91.49	2.53	2	0
Protocatechuic acid	1.09	−1.89	154.12	4	3	37.45	77.76	1.99	1	0
SinocrassosideA1	3.36	−4.09	316.43	3	0	92.90	38.83	2.58	0	0
Acarbose	8.56	−2.13	645.60	19	14	136.69	321.17	2.32	9	3

MW: molecular weight, logP: partition coefficient, tPSA: topological polar surface area, logSw: water solubility, HBA: hydrogen bond acceptors, HBD: hydrogen bond donors.

**Table 3:** ADMET properties of the AAHPE compounds predicted using the SwissADME online analyzer and ADMETlab web server.

Class	Properties	3-Caffeoylquinic Acid	3-*O*-Caffeoyl-4-*O*-Methylquinic Acid	4-/5-Feruloyl Quinic Acid	SinocrassosideA1	Engeletin	Metformin	Protocatechuic Acid	Acarbose	Arbutin
**Absorption**	Caco-2 permeability (˃−5.15 cm/s)	−6.58	−6.331	−6.331	−4.56	−6.581	−5.502	−5.107	−0.8955	−5.954
	Pgp-inhibitor	No	No	No	No	No	No	No	Yes	No
	Pgp-substrate	No	Yes	No	Yes	Yes	No	No	No	No
	HIA (≥30%: high, <30%: low)	Low	High	High	High	High	High	Low	Low	High
	Bioavailability score	0.11	0.11	0.11	0.55	0.55	0.55	0.56	0.11	0.55
	GI absorption	Low	Low	Low	High	Low	High	High	Low	High
	Skin permeation (Log Kp) (cm/s)	−8.76	−8.62	−8.62	−5.65	−8.42	−7.99	−6.39	−5.16	−8.92
**Distribution**	PPB (90%)	41.961%	41.1349%	41.1349%	97.16.5%	42.5742%	3.9577%	42.6641%	21.138%	36.0499%
	BBB	No	No	No	Yes	No	No	No	No	No
**Metabolism**	CYP1A2 inhibitor	No	No	No	No	No	No	No	No	No
	CYP1A2 substrate	No	No	No	No	No	No	No	Yes	No
	CYP3A4 inhibitor	No	No	No	No	No	No	No	No	No
	CYP3A4 substrate	Weakly	Weakly	Weakly	Yes	Weakly	No	Yes	Yes	Weakly
	CYP2C9 inhibitor	No	No	No	Yes	No	No	No	No	No
	CYP2C9 substrate	No	No	No	No	No	No	No	No	No
	CYP2C19 inhibitor	No	No	No	Yes	No	No	No	No	No
	CYP2C19 substrate	No	No	No	No	No	No	No	No	No
	CYP2D6 inhibitor	No	No	No	No	No	No	No	Yes	No
	CYP2D6 substrate	No	No	No	No	No	Yes	No	Weakly	No
**Excretion**	T_1/2_ (˃8 h: high; 3 h < Cl < 8 h: moderate; <3 h: low)	0.442	0.565	0.565	1.587	1.213	1.838	0.318	1.32	0.713
	Clearance rate (˃15 mL/min/kg: high; 5mL/min/kg < Cl < ˃15 mL/min/kg: moderate; <5 mL/ min/kg: low)	1.196	1.174	1.174	1.569	1.033	0.911	1.601	0.503	1.526
**Toxicity**	hERG I/II	No/No	No/No	No/No	No/No	No/No	Yes/Yes	No/No	Ambiguous	No/No
	AMES toxicity	No	No	No	No	No	No	No	No	No
	H-HT (Human hepatotoxicity)	No	No	No	No	No	No	No	No	No
	Skin sensitization	No	No	No	No	No	Yes	No	No	No
	Max. tolerated dose (human) (log mg/kg/day)	−0.134	1.285	1.285	−0.078	0.306	0.902	0.787	0.484	0.485

**Table 4:** Predicted binding affinity and detailed docking interactions of α-amylase and α-glucosidase with compounds of AAHPE and acarbose.

Compounds	Binding Affinity (Kcal/mol)α-Amylase	No of H-Bonds	H-Bonds Residues with H-Bonds Length (Å)	Binding Affinity (Kcal/mol)α-glucosidase	No of H-Bonds	H-Bonds Residues with H-Bonds Length (Å)
3-caffeoylquinic acid	−7.2	4	Ala106 (3.20 Å), Asn105 (3.16 Å), Thr163 (2.65 Å), Gln63 (3.16 Å)	−7.8	6	Gln839 (1.96 Å), Ser774 (2.55 Å), Asn797 (2.68 Å), Thr769 (1.83 Å), Arg773 (2.39 Å) Arg392 (2.78 Å)
3-*O*-Caffeoyl-4-*O*-methylquinic acid	−7.4	5	Asp300 (2.27 Å), Thr163 (3.27 Å), Glu233 (2.13 Å), Asp197 (2.94 Å), His305 (2.08 Å)	−7.6	3	Asn797 (2.48 Å), Thr769 (3.15 Å), Arg392 (2.89 Å)
4-Feruloylquinic acid	−7.7	4	Arg195 (3.20 Å), Gln63 (2.70 Å), Thr163 (2.79 Å), Asp300 (2.24 Å)	−7.3	4	Gln839 (2.70 Å), Trp841 (3.25 Å), Thr769 (2.62 Å), Arg392 (2.98 Å)
5-Feruloylquinic acid	−7.7	2	Glu233 (2.59 Å), Asp197 (2.31 Å)	−7.3	4	Gly390 (2.50 Å), Ser774 (2.98 Å), Trp841 (2.88 Å), Arg392 (2.98 Å)
Arbutin	−7.0	3	Glu233 (2.53 Å), Asp197 (2.23 Å), Gln63 (2.96 Å)	−6.8	5	Thr769 (2.71 Å), Trp320 (3.34 Å), Asn797 (2.79 Å), Arg392 (3.28 Å), His387 (2.77 Å)
Engeletin	−8.5	4	Glu233 (2.63 Å), Arg195 (3.34 Å), Gln63 (2.79 Å), His305 (1.97 Å)	−8.4	5	Arg392 (2.83 Å), Tyr319 (3.13 Å), Trp320 (2.91 Å), Arg773 (3.32 Å), Gln770(2.84 Å)
Acarbose	−6.1	4	Glu233 (3.08 Å) Asp300 (2.49 Å), Thr163 (2.93 Å), Gln63 (2.04 Å)	–6.3	4	Glu109 (2.75 Å), Lys560 (2.19 Å), Thr561 (2.29 Å), Met801 (2.29 Å)
Protocatechuic acid	−7.2	3	Glu233 (2.97 Å), Gln63 (3.06 Å), Thr163 (2.57 Å)	−6.6	3	Thr775 (2.65 Å), Ser774 (2.55 Å), Arg773 (2.79 Å)
SinocrassosideA1	−9.6	3	Gln63 (2.50 Å), Asp300 (2.49 Å), Glu233 (2.27 Å)	−9.0	4	Gln839 (2.22 Å), Ser774 (3.08 Å), Glu352 (2.55 Å), Gly390 (1.99 Å)

## Data Availability

The funders had no role in the design of the study; in the collection, analyses, or interpretation of data; in the writing of the manuscript, or in the decision to publish the results.

## Supplementary Materials

The following are available online, Table S1. LC-MS analysis of bioactive constituents of aqueous-acetone extract of *Helichrysum petiolare* (AAHPE), Table S2. Chemical structure (2D) of bioactive compounds in the aqueous-acetone extract of *Helichrysum petiolare* (AAHPE), Table S3. Predicted binding affinity and detailed docking interactions of α-amylase and α-glucosidase with compounds of AAHPE and Acarbose, Figure S1. Model of the Interaction and the 2D Structure of α-amylase protein and compounds of AAHPE, Figure S2. Model of the Interaction and the 2D Structure of α- glucosidase protein and compounds of AAHPE.

## References

[1] Al-Khafajy D.A., Majeed M.J., Al-Azzawi O.F., Khaleel A.I. (2021). Role of CoQ10 and IGFBP-1 in Obese Male Patients with Diabetic Mellitus Type II. Indian J. Forensic Med. Toxicol..

[2] Hu Y., Hou Z., Liu D., Yang X. (2016). Tartary buckwheat flavonoids protect hepatic cells against high glucose-induced oxidative stress and insulin resistance via MAPK signaling pathways. Food Funct..

[3] Vanessa Fiorentino T., Prioletta A., Zuo P., Folli F. (2013). Hyperglycemia-induced oxidative stress and its role in diabetes mellitus related cardiovascular diseases. Curr. Pharm. Des..

[4] Adrar N.S., Madani K., Adrar S. (2019). Impact of the inhibition of proteins activities and the chemical aspect of polyphenols-proteins interactions. PharmaNutrition.

[5] Wang S., Noh S.K., Koo S.I. (2006). Green tea catechins inhibit pancreatic phospholipase A2 and intestinal absorption of lipids in ovariectomized rats. J. Nutr. Biochem..

[6] Suzuki-Sugihara N., Kishimoto Y., Saita E., Taguchi C., Kobayashi M., Ichitani M., Ukawa Y., Sagesaka Y.M., Suzuki E., Kondo K. (2016). Green tea catechins prevent low-density lipoprotein oxidation via their accumulation in low-density lipoprotein particles in humans. Nutr. Res..

[7] Sun L., Warren F.J., Gidley M.J. (2018). Soluble polysaccharides reduce binding and inhibitory activity of tea polyphenols against porcine pancreatic α-amylase. Food Hydrocoll..

[8] Karas D., Ulrichová J., Valentová K. (2017). Galloylation of polyphenols alters their biological activity. Food Chem. Toxicol..

[9] Omar S.H. (2017). Biophenols pharmacology against the amyloidogenic activity in Alzheimer’s disease. Biomed. Pharmacother..

[10] Miltonprabu S., Tomczyk M., Skalicka-Woźniak K., Rastrelli L., Daglia M., Nabavi S.F., Alavian S.M., Nabavi S.M. (2017). Hepatoprotective effect of quercetin: From chemistry to medicine. Food Chem. Toxicol..

[11] Foegeding E.A., Plundrich N., Schneider M., Campbell C., Lila M.A. (2017). Protein-polyphenol particles for delivering structural and health functionality. Food Hydrocoll..

[12] Hanhineva K., Törrönen R., Bondia-Pons I., Pekkinen J., Kolehmainen M., Mykkänen H., Poutanen K. (2010). Impact of dietary polyphenols on carbohydrate metabolism. Int. J. Mol. Sci..

[13] Ahmed D., Kumar V., Sharma M., Verma A. (2014). Target guided isolation, in-vitro antidiabetic, antioxidant activity and molecular docking studies of some flavonoids from Albizzia Lebbeck Benth. bark. BMC Complementary Altern. Med..

[14] Dai T., Chen J., McClements D.J., Li T., Liu C. (2019). Investigation the interaction between procyanidin dimer and α-glucosidase: Spectroscopic analyses and molecular docking simulation. Int. J. Biol. Macromol..

[15] Sun L., Miao M. (2020). Dietary polyphenols modulate starch digestion and glycaemic level: A review. Crit. Rev. Food Sci. Nutr..

[16] Stratton C.F., Newman D.J., Tan D.S. (2015). Cheminformatic comparison of approved drugs from natural product versus synthetic origins. Bioorganic Med. Chem. Lett..

[17] Segneanu A.E., Velciov S.M., Olariu S., Cziple F., Damian D., Grozescu I., Asao T. (2017). Bioactive Molecules Profile from Natural Compounds. Amino Acid—New Insights and Roles in Plant and Animal.

[18] Akinyede K.A., Cupido C.N., Hughes G.D., Oguntibeju O.O., Ekpo O.E. (2021). Medicinal Properties and In Vitro Biological Activities of Selected Helichrysum Species from South Africa: A Review. Plants.

[19] Albayrak S., Aksoy A., Sagdic O., Hamzaoglu E. (2010). Compositions, antioxidant and antimicrobial activities of Helichrysum (Asteraceae) species collected from Turkey. Food Chem..

[20] Lourens A., Viljoen A.M., Van Heerden F. (2008). South African Helichrysum species: A review of the traditional uses, biological activity and phytochemistry. J. Ethnopharmacol..

[21] Süzgeç-Selçuk S., Birteksöz A. (2011). Flavonoids of Helichrysum chasmolycicum and its antioxidant and antimicrobial activities. S. Afr. J. Bot..

[22] Serabele K., Chen W., Tankeu S., Combrinck S., Veale C.G., van Vuuren S., Chaudhary S.K., Viljoen A. (2021). Comparative chemical profiling and antimicrobial activity of two interchangeably used ‘Imphepho’species (*Helichrysum odoratissimum* and *Helichrysum petiolare*). S. Afr. J. Bot..

[23] Lourens A., Van Vuuren S., Viljoen A., Davids H., Van Heerden F. (2011). Antimicrobial activity and in vitro cytotoxicity of selected South African Helichrysum species. S. Afr. J. Bot..

[24] Odeyemi S., Bradley G. (2018). Medicinal plants used for the traditional management of diabetes in the Eastern Cape, South Africa: Pharmacology and toxicology. Molecules.

[25] Yildirim B.A., Kordali S., Kapakin K.A.T., Yildirim F., Senocak E.A., Altun S. (2017). Effect of Helichrysum plicatum DC. subsp. plicatum ethanol extract on gentamicin-induced nephrotoxicity in rats. J. Zhejiang Univ.-Sci. B.

[26] Aslan M., Orhan D.D., Orhan N., Sezik E., Yeşilada E. (2007). A study of antidiabetic and antioxidant effects of Helichrysum graveolens capitulums in streptozotocin-induced diabetic rats. J. Med. Food.

[27] Nasr A., Zhou X., Liu T., Yang J., Zhu G.-P. (2019). Acetone-water mixture is a competent solvent to extract phenolics and antioxidants from four organs of Eucalyptus camaldulensis. Turk. J. Biochem..

[28] Ali H., Houghton P., Soumyanath A. (2006). α-Amylase inhibitory activity of some Malaysian plants used to treat diabetes; with particular reference to Phyllanthus amarus. J. Ethnopharmacol..

[29] Matsui T., Ueda T., Oki T., Sugita K., Terahara N., Matsumoto K. (2001). α-Glucosidase inhibitory action of natural acylated anthocyanins. 1. Survey of natural pigments with potent inhibitory activity. J. Agric. Food Chem..

[30] Stander M.A., Van Wyk B.-E., Taylor M.J., Long H.S. (2017). Analysis of phenolic compounds in rooibos tea (*Aspalathus linearis*) with a comparison of flavonoid-based compounds in natural populations of plants from different regions. J. Agric. Food Chem..

[31] Oselusi S.O., Christoffels A., Egieyeh S.A. (2021). Cheminformatic Characterization of Natural Antimicrobial Products for the Development of New Lead Compounds. Molecules.

[32] Daina A., Michielin O., Zoete V. (2017). SwissADME: A free web tool to evaluate pharmacokinetics, drug-likeness and medicinal chemistry friendliness of small molecules. Sci. Rep..

[33] Bolton E.E., Wang Y., Thiessen P.A., Bryant S.H. (2008). PubChem: Integrated Platform of Small Molecules and Biological Activities. Annual Reports in Computational Chemistry.

[34] Williams A.J. (2008). Public chemical compound databases. Curr. Opin. Drug Discov. Dev..

[35] Oyewusi H.A., Huyop F., Wahab R.A. (2020). Molecular docking and molecular dynamics simulation of Bacillus thuringiensis dehalogenase against haloacids, haloacetates and chlorpyrifos. J. Biomol. Struct. Dyn..

[36] Oyewusi H.A., Huyop F., Wahab R.A., Hamid A.A.A. (2021). In silico assessment of dehalogenase from *Bacillus thuringiensis* H2 in relation to its salinity-stability and pollutants degradation. J. Biomol. Struct. Dyn..

[37] Kidane Y., Bokrezion T., Mebrahtu J., Mehari M., Gebreab Y.B., Fessehaye N., Achila O.O. (2018). In vitro inhibition of-amylase and-glucosidase by extracts from Psiadia punctulata and *Meriandra bengalensis*. Evid.-Based Complement. Altern. Med..

[38] Atchan Nwakiban A.P., Cicolari S., Piazza S., Gelmini F., Sangiovanni E., Martinelli G., Bossi L., Carpentier-Maguire E., Deutou Tchamgoue A., Agbor G.A. (2020). Oxidative stress modulation by cameroonian spice extracts in hepg2 cells: Involvement of nrf2 and improvement of glucose uptake. Metabolites.

[39] Lipinski C.A., Lombardo F., Dominy B.W., Feeney P.J. (1997). Experimental and computational approaches to estimate solubility and permeability in drug discovery and development settings. Adv. Drug Deliv. Rev..

[40] Oselusi S.O., Egieyeh S.A., Christoffels A. (2021). Cheminformatic Profiling and Hit Prioritization of Natural Products with Activities against Methicillin-Resistant Staphylococcus aureus (MRSA). Molecules.

[41] Tian S., Wang J., Li Y., Li D., Xu L., Hou T. (2015). The application of in silico drug-likeness predictions in pharmaceutical research. Adv. Drug Deliv. Rev..

[42] Kirchmair J., Göller A.H., Lang D., Kunze J., Testa B., Wilson I.D., Glen R.C., Schneider G. (2015). Predicting drug metabolism: Experiment and/or computation?. Nat. Rev. Drug Discov..

[43] Patil R., Das S., Stanley A., Yadav L., Sudhakar A., Varma A.K. (2010). Optimized hydrophobic interactions and hydrogen bonding at the target-ligand interface leads the pathways of drug-designing. PLoS ONE.

[44] Verma M., Gupta S.J., Chaudhary A., Garg V.K. (2017). Protein tyrosine phosphatase 1B inhibitors as antidiabetic agents—A brief review. Bioorg. Chem..

[45] Wang L.-J., Jiang B., Wu N., Wang S.-Y., Shi D.-Y. (2015). Natural and semisynthetic protein tyrosine phosphatase 1B (PTP1B) inhibitors as anti-diabetic agents. RSC Adv..

[46] Ali M.Y., Jannat S., Jung H.A., Choi J.S. (2021). Insulin–Mimetic Dihydroxanthyletin-Type Coumarins from Angelica decursiva with Protein Tyrosine Phosphatase 1B and α-Glucosidase Inhibitory Activities and Docking Studies of Their Molecular Mechanisms. Antioxidants.

[47] Gray G.M. (1975). Carbohydrate digestion and absorption: Role of the small intestine. N. Engl. J. Med..

[48] Brogi S., Ramalho T.C., Kuca K., Medina-Franco J.L., Valko M. (2020). In silico Methods for Drug Design and Discovery. Front. Chem..

[49] Sun H., Scott D.O. (2010). Structure-based drug metabolism predictions for drug design. Chem. Biol. Drug Des..

[50] Blundell T.L., Sibanda B.L., Montalvão R.W., Brewerton S., Chelliah V., Worth C.L., Harmer N.J., Davies O., Burke D. (2006). Structural biology and bioinformatics in drug design: Opportunities and challenges for target identification and lead discovery. Philos. Trans. R. Soc. B Biol. Sci..

[51] Jhong C.H., Riyaphan J., Lin S.H., Chia Y.C., Weng C.F. (2015). S creening alpha-glucosidase and alpha-amylase inhibitors from natural compounds by molecular docking in silico. Biofactors.

[52] Aladejana A.E., Bradley G., Afolayan A.J. (2020). In vitro evaluation of the anti-diabetic potential of Helichrysum petiolare Hilliard & BL Burtt using HepG2 (C3A) and L6 cell lines. F1000Research.

[53] Fernandez E., Ross C., Liang H., Javors M., Tardif S., Salmon A.B. (2019). Evaluation of the pharmacokinetics of metformin and acarbose in the common marmoset. Pathobiol. Aging Age-Relat. Dis..

[54] Zonoubi A., Prashantha C.N., Perumal D.V., Mafibaniasadi Z. (2019). In silico Analysis of Active Constituents of Silymarin as Alpha-Glucosidase Enzyme Inhibitors in Type 2 Diabetes Mellitus. Asian J. Pharm. Clin. Res..

[55] Oyinloye B.E., Adekiya T.A., Aruleba R.T., Ojo O.A., Ajiboye B.O. (2019). Structure-based docking studies of GLUT4 towards exploring selected phytochemicals from Solanum xanthocarpum as a therapeutic target for the treatment of cancer. Curr. Drug Discov. Technol..

[56] Li Y., Liu X., Zhou H., Li B., Mazurenko I.K. (2021). Inhibitory Mechanism of Engeletin Against α-Glucosidase. Natural Product Communications.

[57] Yousefi F., Mahjoub S., Pouramir M., Khadir F. (2013). Hypoglycemic activity of Pyrus biossieriana Buhse leaf extract and arbutin: Inhibitory effects on alpha amylase and alpha glucosidase. Casp. J. Intern. Med..

[58] Floris S., Fais A., Medda R., Pintus F., Piras A., Kumar A., Kuś P.M., Westermark G.T., Era B. (2021). Washingtonia filifera seed extracts inhibit the islet amyloid polypeptide fibrils formations and α-amylase and α-glucosidase activity. J. Enzym. Inhib. Med. Chem..

[59] Blakaj D.M., McConnell K.J., Beveridge D.L., Baranger A.M. (2001). Molecular dynamics and thermodynamics of protein−RNA interactions: Mutation of a conserved aromatic residue modifies stacking interactions and structural adaptation in the U1A−stem loop 2 RNA complex. J. Am. Chem. Soc..

[60] Pecsi I., Leveles I., Harmat V., Vertessy B.G., Toth J. (2010). Aromatic stacking between nucleobase and enzyme promotes phosphate ester hydrolysis in dUTPase. Nucleic Acids Res..

[61] Lo Piparo E., Scheib H., Frei N., Williamson G., Grigorov M., Chou C.J. (2008). Flavonoids for controlling starch digestion: Structural requirements for inhibiting human α-amylase. J. Med. Chem..

[62] Bano S., Khan A.-u., Asghar F., Usman M., Badshah A., Ali S. (2018). Computational and pharmacological evaluation of Ferrocene-based acyl ureas and homoleptic cadmium carboxylate derivatives for anti-diabetic potential. Front. Pharmacol..

[63] Ahmed M.S., Khan A.-u., Kury L.T.A., Shah F.A. (2020). Computational and Pharmacological Evaluation of Carveol for Antidiabetic Potential. Front. Pharmacol..

[64] Abdel-Mageid A.D., Abou-Salem M.E.S., Salaam N.M.H.A., El-Garhy H.A.S. (2018). The potential effect of garlic extract and curcumin nanoparticles against complication accompanied with experimentally induced diabetes in rats. Phytomedicine.

[65] Safitri A., Sari D.R.T., Fatchiyah F., Roosdiana A. (2021). Modeling of Aqueous Root Extract Compounds of Ruellia tuberosa L. for Alpha-Glucosidase Inhibition Through in Silico Study. Makara J. Sci..

