# Supplementary material for: In Vitro Evaluation of the Anti-Diabetic Potential of Aqueous Acetone Helichrysum petiolare Extract (AAHPE) with Molecular Docking Relevance in Diabetes Mellitus
Source: Molecules. 2021 Dec 28;27(1):155. doi: 10.3390/molecules27010155 (PMC8746515; doi:10.3390/molecules27010155)
Supplement: Supplementary file 1 [file molecules-27-00155-s001.zip › molecules-1455582-supplementary.pdf]

Table S1: LC-MS analysis of bioactive constituents of aqueous-acetone extract of *Helichrysum petiolare* (AAHPE)

Compound	Name	Average R_t(min)	Average *m/z*	Concentration(mg/g)
1	Arbutin	5,475	271,08157	1279,2
2	Protocatechuic acid 4-O-glucoside	7,913	315,06607	182,9
3	3-caffeoylquinic acid (neochlorogenic acid)	9,609	353,09525	120,2
4	Chlorogenic acid (5-caffeoylquinic acid)	11,975	353,0864	826,7
5	Caffeic acid derivative	12,758	459,1528	134,4
6	UNPD8659	13,638	349,05746	131,8
7	Dactylin	14,234	639,1543	55,0
8	1,3-Dicaffeoylquinic acid	14,433	515,11609	388,7
9	UNPD204949	14,769	377,1803	78,6
10	UNPD114029	14,968	423,09171	167,4
11	quercetin-3-O-(feruloyl) sophoroside	15,203	801,18866	71,6
12	5-Feruloyl quinic acid	15,296	367,10284	411,6
13	3-O-p-Coumaroylquinic acid	15,54	337,09311	82,7
14	Sakuranin	15,562	447,12833	3,5
15	Myricetin 3-galactoside	15,779	479,08148	402,5
16	4-Feruloyl quinic acid	16,016	367,10156	363,2
17	Quercetin 3-galactoside	16,285	463,0874	586,3
18	3-O-Caffeoyl-4-O-methylquinic acid	16,515	367,10147	407,1
19	Sinapic acid	17,052	223,05902	14,9

20	Engeletin	17,334	433,11368	749,7
21	Rutin	17,426	609,14587	12,8
22	Quercetin 3-glucoside	17,656	463,08673	118,5
23	Kaempferol galactoside (Trifolin)	18,06	447,09433	166,2
24	UNPD80025	18,352	493,09372	104,2
25	1,4-Dicaffeoylquinic acid	18,633	515,11823	258,9
26	3,4-Dicaffeoylquinic acid	18,797	515,11768	535,3
27	3,5-Dicaffeoylquinic acid	19,236	515,12036	1727,3
28	Kaempferol glucoside (Astragalin)	20,078	447,09393	13,4
29	4,5-Dicaffeoylquinic acid	20,34	515,11981	1209,1
30	Sakuranetin	20,927	285,07538	6,1
31	Sinocrassoside A1	21,036	501,13861	127,1
32	Cascaroside C	21,652	563,17737	116,3
33	Feruloyl quinic acid	22,12	367,10077	120,5
34	3,5-Dicaffeoylquinic methyl ester	23,102	529,13446	319,0
35	Sakuranin isomer	23,403	447,1297	4,5
36	Kaempferol	24,086	285,04065	27,1
37	Luteolin	24,32	531,15051	1,9
38	Sakuranetin isomer	24,447	285,07715	2,0

Table S2: Chemical structure (2D) of bioactive compounds in the aqueous-acetone extract of *Helichrysum petiolare* (AAHPE)

Bioactive compounds	PubChem CID/ChemSpider ID	Molecular formula	Calonical SMILES	Chemical structure
1,3-Dicaffeoylquinic acid	6474640	$C_{25}H_{24}O_{12}$	C1C(C(C(CC1(C(=O)O)OC(=O)C=CC2=CC(=C(C=C2)O)O)OC(=O)C=CC3=CC(=C(C=C3)O)O)O)O	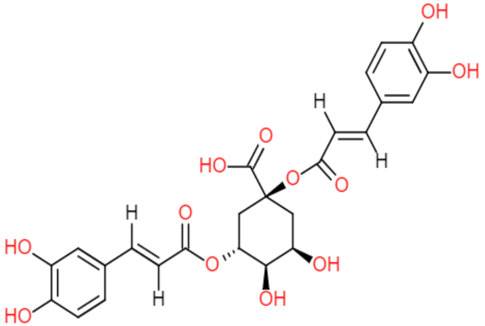
1,4-Dicaffeoylquinic acid	12358846	$C_{25}H_{24}O_{12}$	C1C(C(C(CC1(C(=O)O)OC(=O)C=CC2=CC(=C(C=C2)O)O)O)OC(=O)C=CC3=CC(=C(C=C3)O)O)O	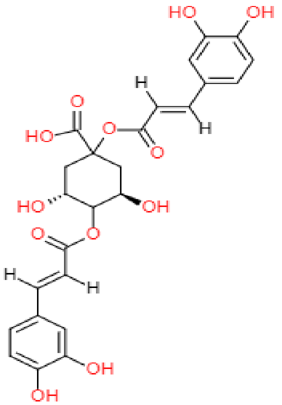

3,4-Dicaffeoylquinic acid	5281780	$C_{25}H_{24}O_{12}$	C1C(C(C(CC1(C(=O)O)O)OC(=O)C=CC2=CC(=C(C=C2)O)O)OC(=O)C=CC3=CC(=C(C=C3)O)O)O	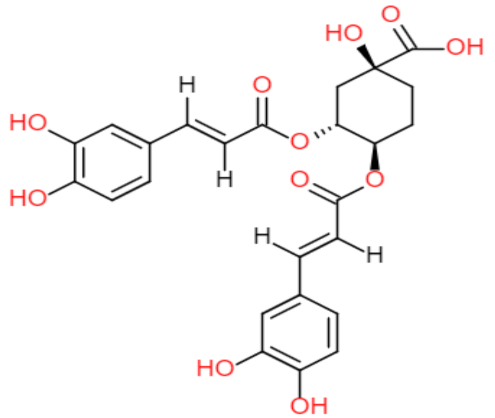
3,5-Dicaffeoylquinic acid	6474310	$C_{25}H_{24}O_{12}$	C1C(C[CH](C([CH]1OC(=O)/C=C/C2=CC(=C(C=C2)O)O)O)OC(=O)/C=C/C3=CC(=C(C=C3)O)O)(O)C(=O)O	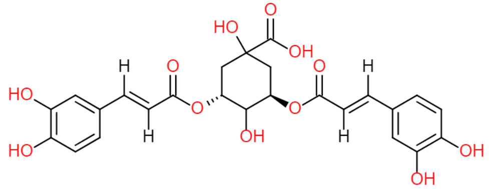
3-caffeoylquinic acid	1794427	$C_{16}H_{18}O_9$	C1C(C(C(CC1(C(=O)O)O)OC(=O)C=CC2=CC(=C(C=C2)O)O)O)O	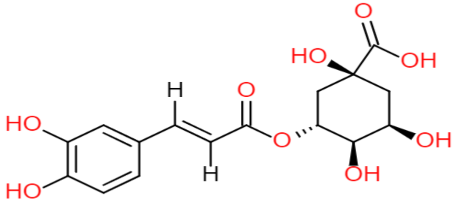
3-O-Caffeoyl-4-O-methylquinic acid	131752769	$C_{17}H_{20}O_9$	COC1=C(C=CC(=C1)C=CC(=O)OC2CC(CC(C2O)O)(C(=O)O)O)O	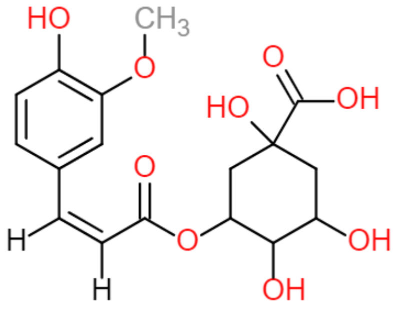

4,5-Dicaffeoylquinic acid	6474309	$C_{25}H_{24}O_{12}$	C1C(C(C(CC1(C(=O)O)O)OC(=O)C=CC2=CC(=C(C=C2)O)O)OC(=O)C=CC3=CC(=C(C=C3)O)O)O	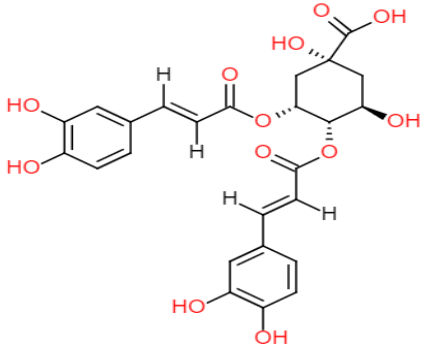
3-Feruloyl quinic acid/5-Feruloylquinic acid	73210496	$C_{17}H_{20}O_9$	COC1=C(C=CC(=C1)C=CC(=O)OC2CC(CC(C2O)O)(C(=O)O)O)O	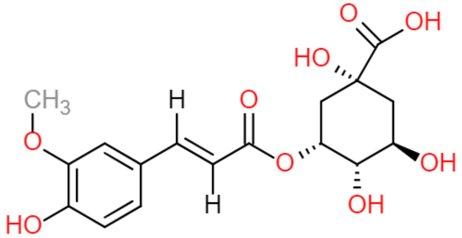
Arbutin	440936	$C_{12}H_{16}O_7$	C1=CC(=CC=C1O)OC2C(C(C(C(O2)CO)O)O)O	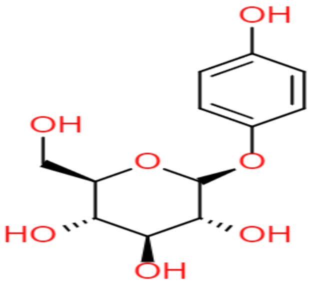

Cascaroside C	46173832	$C_{27}H_{32}O_{13}$	CC1=CC2=C(C(C(=C1)O)C(=O)C3=C(C2C4C(C(C(C(O4)CO)O)O)O)C=CC=C3OC5C(C(C(C(O5)CO)O)O)O)O	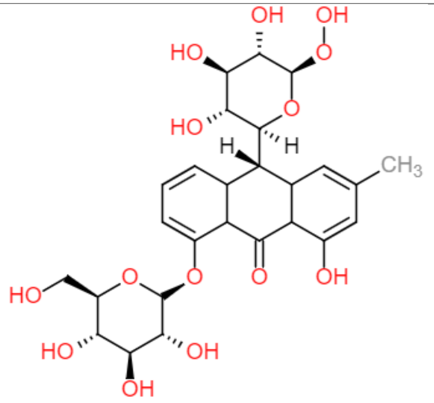
Engeletin	6453452	$C_{21}H_{22}O_{10}$	CC1C(C(C(C(O1)OC2C(OC3=CC(=CC(=C3C2=O)O)O)C4=CC=C(C=C4)O)O)O)O	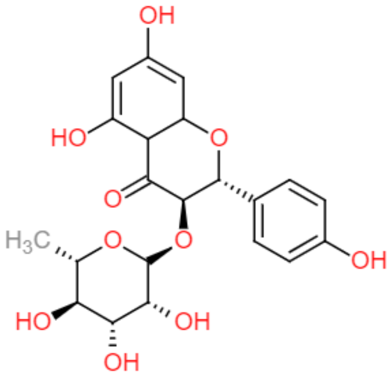
Isorhamnetin 3-galactoside	13245586	$C_{22}H_{22}O_{12}$	COC1=C(C=CC(=C1)C2=C(C(=O)C3=C(C=C(C=C3O2)O)O)OC4C(C(C(C(O4)CO)O)O)O)O	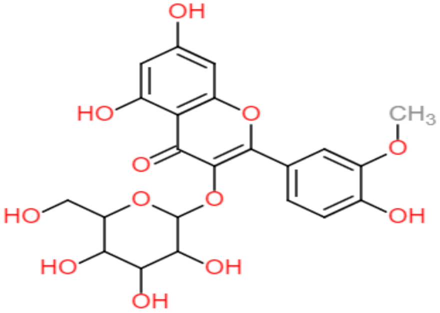

Kaempferol galactoside (Trifolin)	5282149	$C_{21}H_{20}O_{11}$	C1=CC(=CC=C1C2=C(C(=O)C3=C(C=C(C=C3O2)O)O)OC4C(C(C(C(O4)CO)O)O)O)O	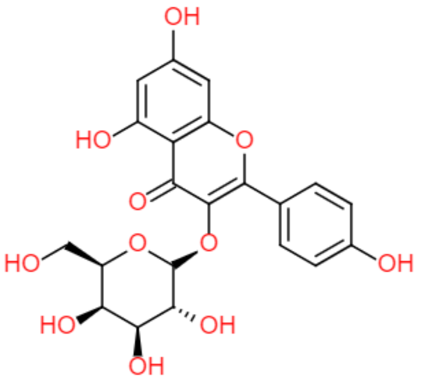
Metformin	4091	$C_{4}H_{11}N_{5}$	CN(C)C(=N)N=C(N)N	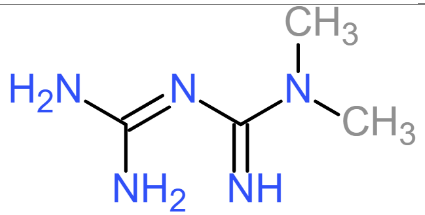
Acarbose	41774	$C_{25}H_{43}NO_{18}$	CC1C(C(C(C(O1)OC2C(OC(C(C2O)O)OC3C(OC(C(C3O)O)O)CO)CO)O)O)NC4C=C(C(C(C4O)O)O)CO	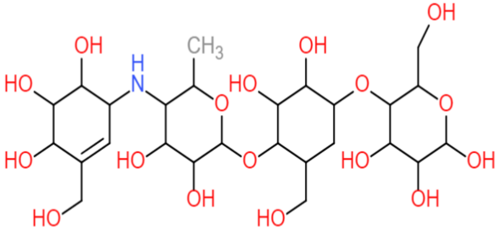
Methyl 3,5-di-O-caffeoyl quinate	10075681	$C_{26}H_{26}O_{12}$	COC(=O)C1(CC(C(C(C1)OC(=O)C=CC2=CC(=C(C=C2)O)O)O)OC(=O)C=CC3=CC(=C(C=C3)O)O)O	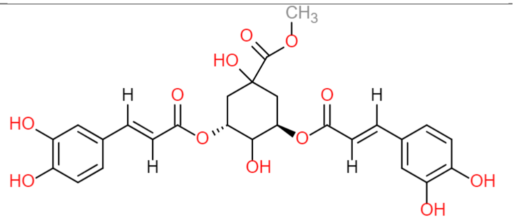

Myricetin 3-galactoside	5491408	$C_{21}H_{20}O_{13}$	C1=C(C=C(C(=C1O)O)O)C2=C(C(=O)C3=C(C=C(C=C3O2)O)O)OC4C(C(C(C(O4)CO)O)O)O	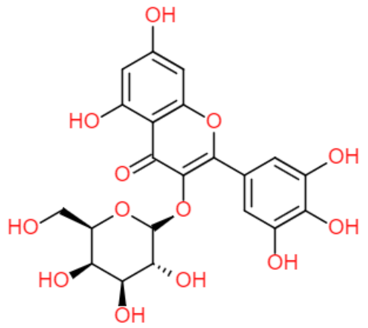
Protocatechuic acid 4-0-glucoside	19	$C_7H_6O_4$	C1=CC(=C(C(=C1)O)O)C(=O)O	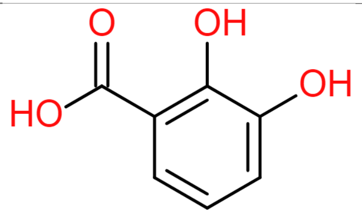
Quercetin 3-galactoside	5281643	$C_{21}H_{20}O_{12}$	C1=CC(=C(C=C1C2=C(C(=O)C3=C(C=C(C=C3O2)O)O)OC4C(C(C(C(O4)CO)O)O)O)O)O	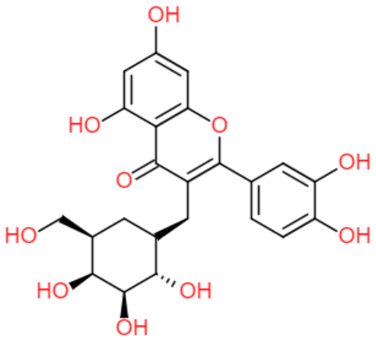

Quercetin 3-glucoside	5280804	$C_{21}H_{20}O_{12}$	C1=CC(=C(C=C1C2=C(C(=O)C3=C(C=C(C=C3O2)O)O)OC4C(C(C(C(O4)CO)O)O)O)O)[O-]	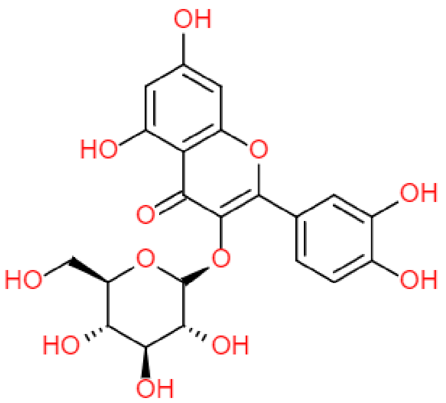
SinocrassosideA1	643719	$C_{20}H_{28}O_3$	CC1=CCCC2(C(O2)CC3C(CC(=CCC1)C)OC(=O)C3=C)C	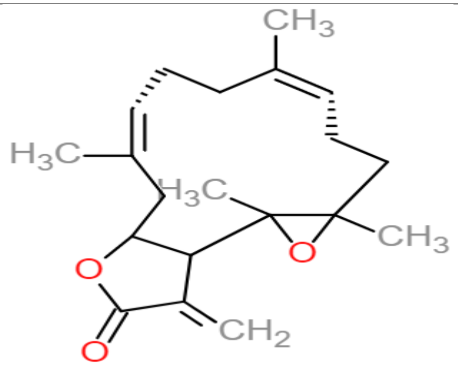

Table S3. Predicted binding affinity and detailed docking interactions of α-amylase and α-glucosidase with compounds of AAHPE *and* Acarbose.

Compounds	Binding affinity (Kcal/mol)	No of H-bonds	H-bonds residues with H-bonds length (Å)	Binding affinity (Kcal/mol)	No of H-bonds	H-bonds residues with H-bonds length (A)
**1,3-Dicaffeoylquinic acid**	– 7.2	2	Asp197 (2.67 Å), Thr163 (2.93 Å)	– 7.6	6	Arg392 (2.99 Å), Thr769 (2.80 Å), Ser774 (2.70 Å), Arg773 (2.56 Å), Gln770 (3.04 Å), Gln839 (2.99 Å)
**1,4-Dicaffeoylquinic acid**	– 8.9	7	Thr163 (2.75 Å), His305 (2.25 Å), Gln63 (3.26 Å), Asp197 (3.30 Å), Glu233 (2.15 Å), His299 (3.02 Å), Asp300 (2.41 Å)	– 9.2	6	Gln839 (2.53 Å), Arg840 (3.02 Å), Val842 (3.53 Å), Try841 (2.21 Å), Arg773 (2.90 Å), Gly390 (2.98 Å)
**3,4-Dicaffeoylquinic acid**	– 8.8	4	Thr163 (3.08 Å), Gln63 (3.08 Å), Asp197 (2.75 Å), Glu233 (2.37 Å)	– 7.9	5	Gln770 (2.22 Å), Ser774 (2.81 Å), Trp841 (3.36 Å), Thr769 (3.04 Å), Arg773 (2.69 Å)
**3,5-Dicaffeoylquinic acid**	– 8.6	4	Thr163 (2.89 Å), Gln63 (1.94 Å), Asp197 (2.61 Å), Glu233 (2.33 Å)	– 8.4	4	Arg552 (3.14 Å), Asp469 (2.47 Å), Asp232 (2.07 Å), Asn496 (2.19 Å)
**4,5-Dicaffeoylquinic acid**	– 8.7	3	Gln63 (2.70 Å), Thr163 (2.91 Å), Glu233 (3.00 Å)	– 8.4	5	Gln839 (3.02 Å), Ser774 (2.21 Å), Gly390 (2.57 Å), Thr769 (3.08 Å), Arg840 (2.98 Å)
**5-caffeoylquinic acid**	– 8.4	6	Asp300 (2.49 Å), Try151 (2.95 Å), Glu233 (2.57 Å), His305 (2.08 Å), His299 (2.46 Å), Gln63 (2.93 Å)	– 7.7	4	Glu545 (3.00 Å), Thr769 (2.83 Å), Glu352 (2.97 Å), Arg392 (2.95 Å)
**Cascaroside C**	– 8.2	3	Glu233 (1.84 Å), Asp197 (2.76 Å), His299 (2.83 Å)	– 7.1	3	Gly770 (3.33 Å), Ser774 (2.83 Å), Arg392 (3.18 Å)
**Isorhamnetin 3-galactoside**	– 8.5	3	Asp300 (2.25 Å), Gln63 (2.91 Å), His305 (2.92 Å)	– 7.8	5	Thr769 (2.14 Å), Ser774 (2.70 Å), Gln770 (2.18 Å), Arg773 (3.40 Å), Asn797 (3.08Å)
**Kaempferol galactoside (Trifolin)**	– 8.2	4	Asp197 (2.76 Å), Asp300 (2.05 Å), Gln63 (3.25 Å), Trp59 (2.35 Å)	– 7.3	4	His387 (2.29 Å), Thr769 (2.25 Å), Trp320 (3.08 Å), Arg392 (2.97 Å)
**Methyl 3,5-di-O-caffeoyl quinate**	– 8.7	6	Glu233 (2.03 Å), Thr163 (2.34 Å), Asp300 (2.93 Å), Gln63 (3.20 Å), His305 (3.17 Å), Trp59 (2.95 Å)	– 8.2	6	Arg388 (2.95 Å), Ser774 (2.87 Å), Thr768 (2.87 Å), Arg392 (3.11 Å), Arg773 (3.20 Å), Arg388 (2.95 Å)
**Myricetin-3-galactoside**	– 8.3	5	His299 (2.48 Å), His305 (3.22 Å), Trp59 (2.36 Å), Gln63 (3.14 Å), Thr163 (2.72 Å)	– 7.9	5	Trp841 (3.02 Å), Arg392 (3.00 Å), Arg773 (3.29 Å), Gln770 (3.29 Å), Gln839 (2.03 Å)
**Quercetin-3-galactoside**	– 8.1	3	His305 (2.13 Å), Asp197 (3.00 Å), His299 (3.09 Å)	– 7.7	5	Trp320 (3.11 Å), Gly798 (2.48 Å), Arg773 (3.24 Å), Gln770 (3.35 Å), Ser774 (2.25 Å)
**Quercetin-3-glucoside**	– 7.8	4	His299 (2.85 Å), Asp197 (2.54 Å), Glu233 (2.56 Å), Arg195 (3.32 Å)	– 7.9	6	Trp320 (3.11 Å), Gln770 (3.35 Å), Ser774 (2.91 Å), Arg773 (3.23 Å), Trp841 (2.93 Å), Gly798 (2.48 Å),

Figure S1. Model of the Interaction and the 2D Structure of α-amylase protein with (a) 1,3-Dicaffeoylquinic acid (b) 1,4-Dicaffeoylquinic acid (c) 3,4-Dicaffeoylquinic acid (d) 3,5-Dicaffeoylquinic acid (e) 4,5-Dicaffeoylquinic acid (f) 5-caffeoylquinic acid (g) Cascaroside C (h) Isorhamnetin 3-galactoside (i) Kaempferol galactoside (Trifolin) (j) Methyl 3,5-di-O-caffeoyl quinate (k) Myricetin-3-galactoside (l) Quercetin-3-galactoside (m) Quercetin-3-glucoside.

a

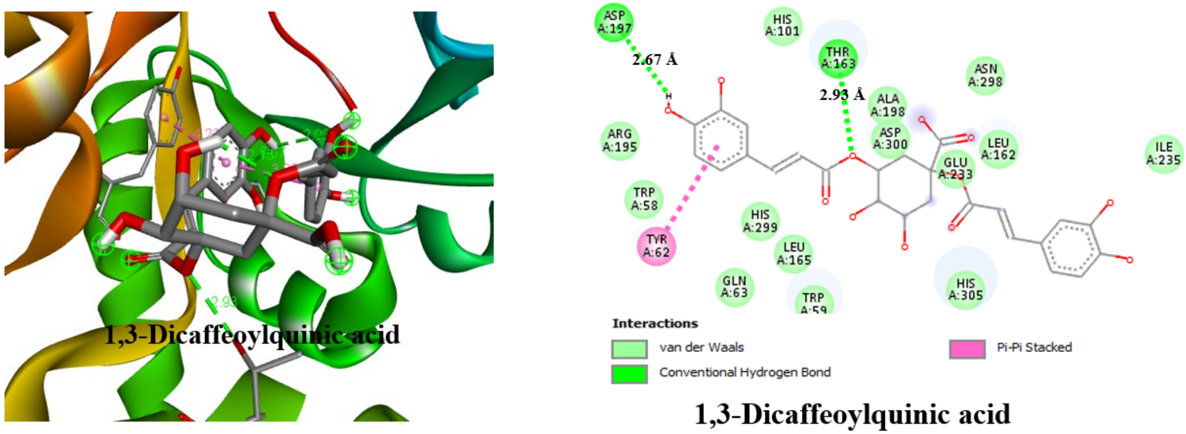

b

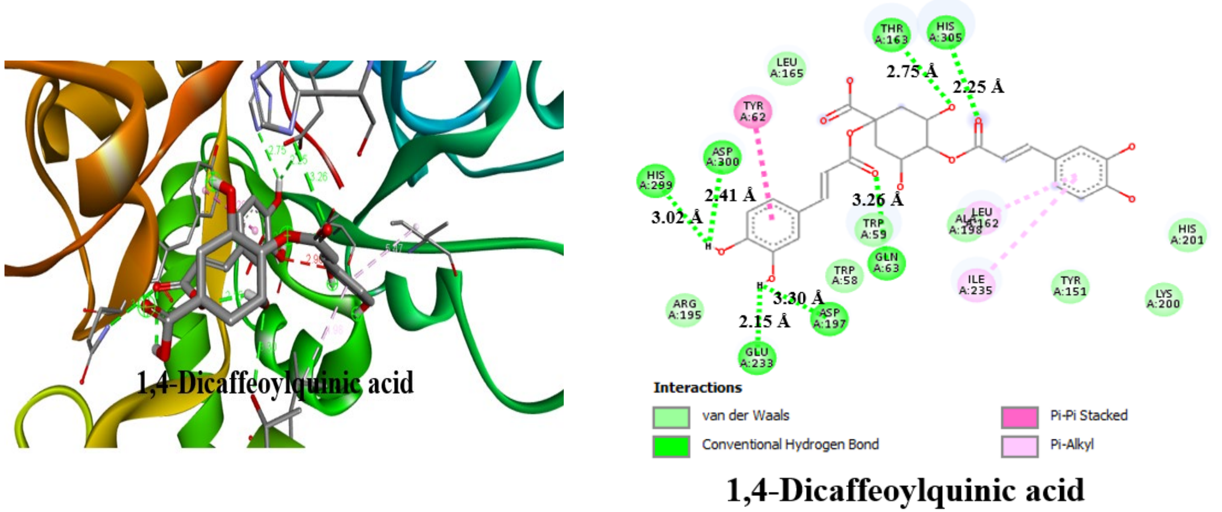

c

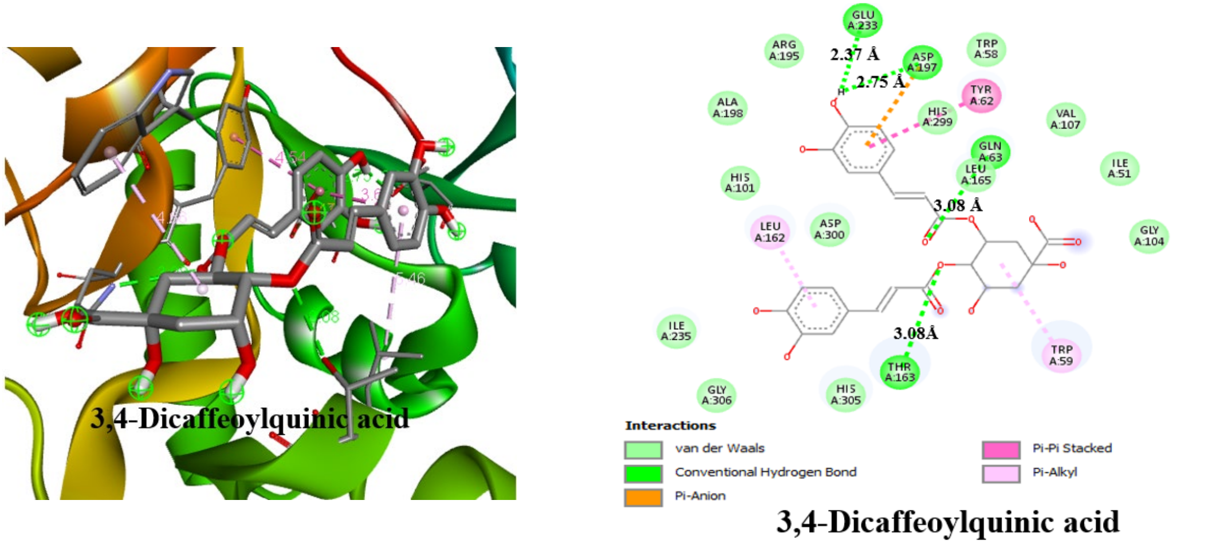
3,4-Dicaffeoylquinic acid
GLU A:233
ARG A:195
ASP A:197
TRP A:58
TYR A:62
ALA A:198
HIS A:299
VAL A:107
GLN A:63
LEU A:165
ILE A:51
HIS A:101
LEU A:162
ASP A:300
GLY A:104
ILE A:235
THR A:163
TRP A:59
GLY A:306
HIS A:305
2.37 Å
2.75 Å
3.08 Å
3.08Å
Interactions
van der Waals
Conventional Hydrogen Bond
Pi-Anion
Pi-Pi Stacked
Pi-Alkyl
3,4-Dicaffeoylquinic acid

d

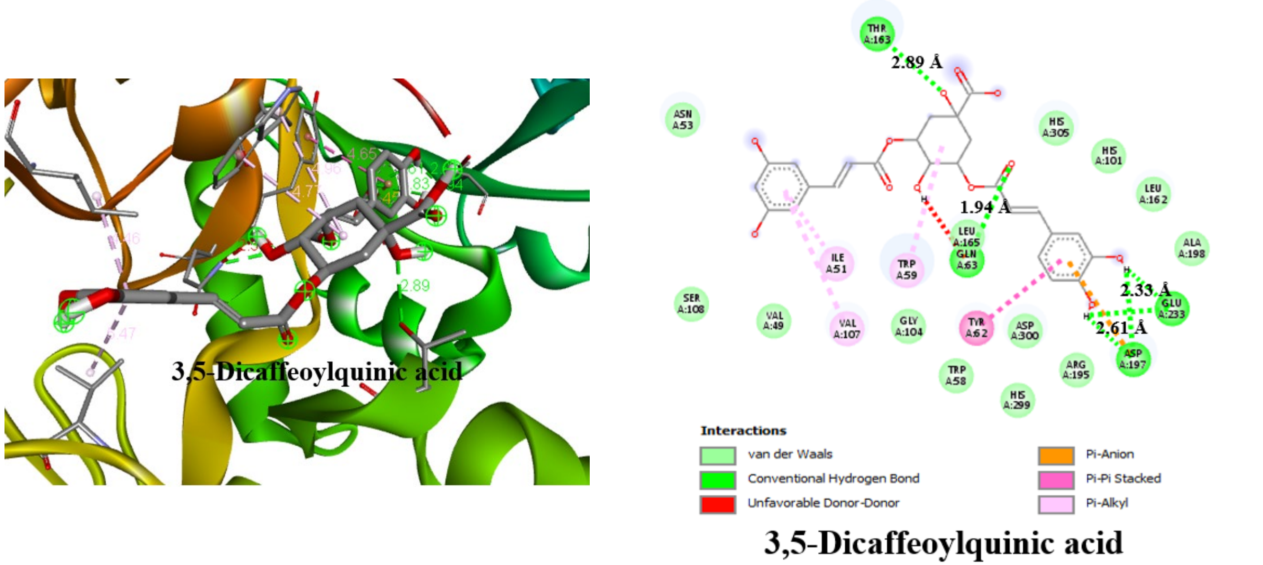
3,5-Dicaffeoylquinic acid
THR A:163
2.89 Å
ASN A:53
HIS A:305
HIS A:101
LEU A:162
1.94 Å
LEU A:165
GLN A:63
ALA A:198
ILE A:51
TRP A:59
2.33 Å
GLU A:233
SER A:108
VAL A:49
VAL A:107
GLY A:104
TYR A:62
ASP A:300
2.61 Å
ASP A:197
ARG A:195
TRP A:58
HIS A:299
Interactions
van der Waals
Conventional Hydrogen Bond
Unfavorable Donor-Donor
Pi-Anion
Pi-Pi Stacked
Pi-Alkyl
3,5-Dicaffeoylquinic acid

e

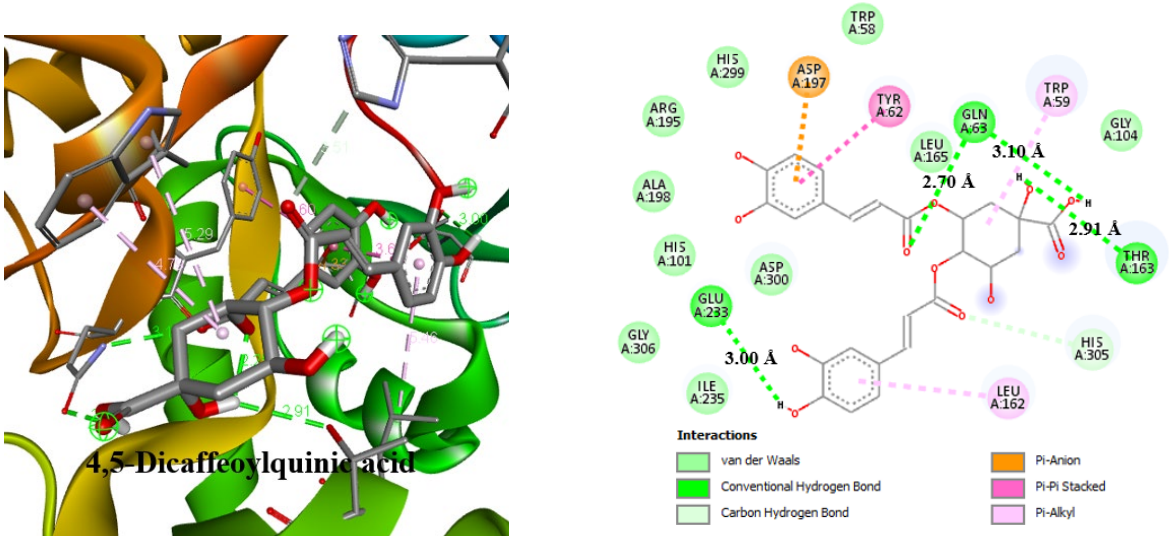

4,5-Dicaffeoylquinic acid

f

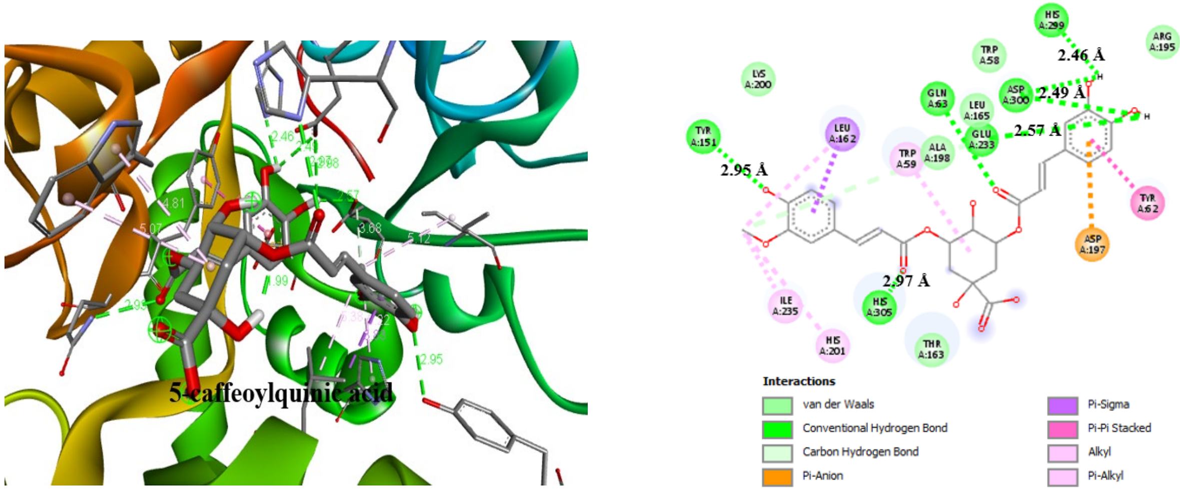

5-caffeoylquinic acid

g

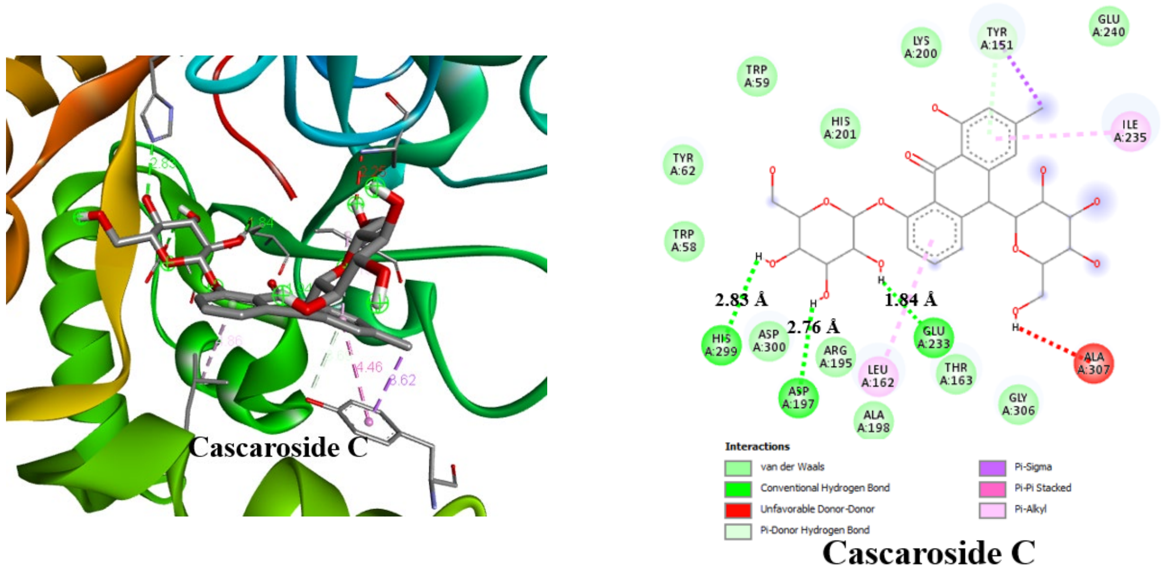
Cascaroside C
TRP A:59
LYS A:200
TYR A:151
GLU A:240
HIS A:201
ILE A:235
TYR A:62
TRP A:58
2.83 Å
2.76 Å
1.84 Å
HIS A:299
ASP A:300
ARG A:195
GLU A:233
LEU A:162
THR A:163
ALA A:307
ASP A:197
GLY A:306
ALA A:198
Interactions
van der Waals
Conventional Hydrogen Bond
Unfavorable Donor-Donor
Pi-Donor Hydrogen Bond
Pi-Sigma
Pi-Pi Stacked
Pi-Alkyl
Cascaroside C

h

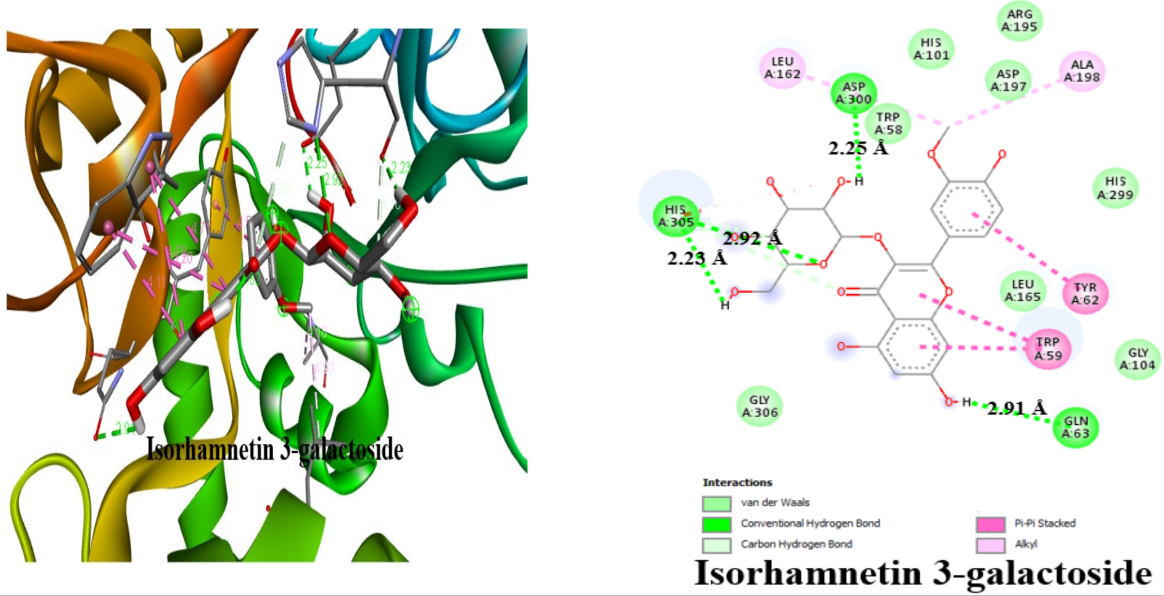
Isorhamnetin 3-galactoside
ARG A:195
LEU A:162
HIS A:101
ASP A:300
ASP A:197
ALA A:198
TRP A:58
2.25 Å
HIS A:299
HIS A:305
2.92 Å
2.23 Å
LEU A:165
TYR A:62
TRP A:59
GLY A:104
GLY A:306
2.91 Å
GLN A:63
Interactions
van der Waals
Conventional Hydrogen Bond
Carbon Hydrogen Bond
Pi-Pi Stacked
Alkyl
Isorhamnetin 3-galactoside

i

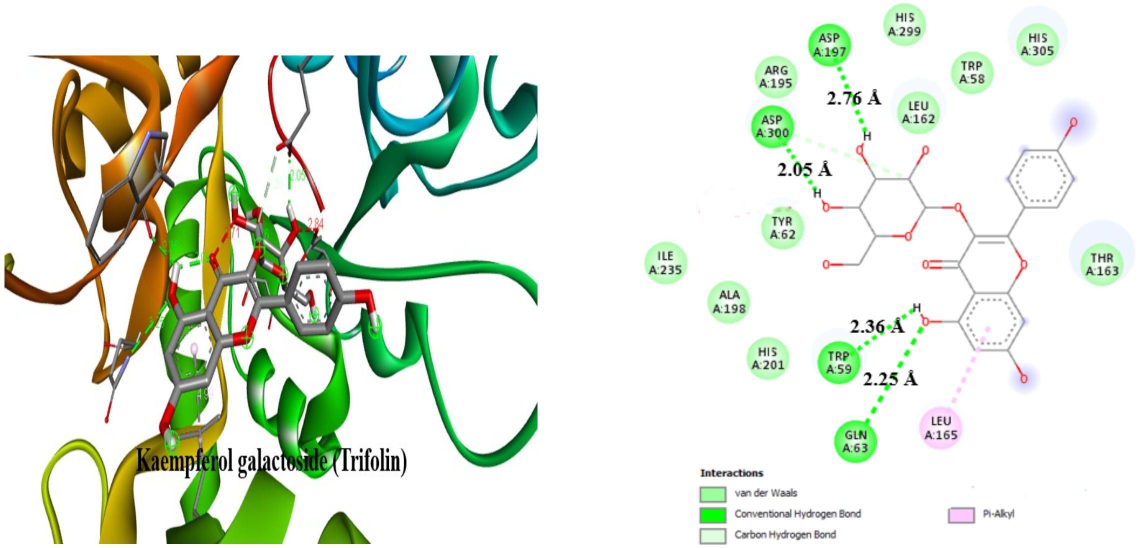

Kaempferol galactoside (Trifolin)

j

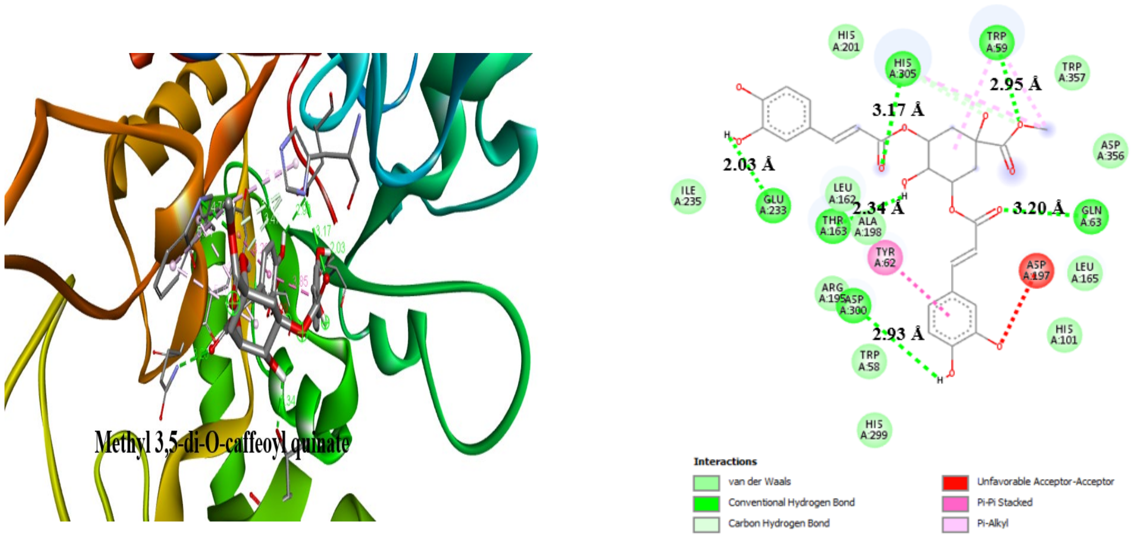

Methyl 3,5-di-O-caffeoyl quinate

k

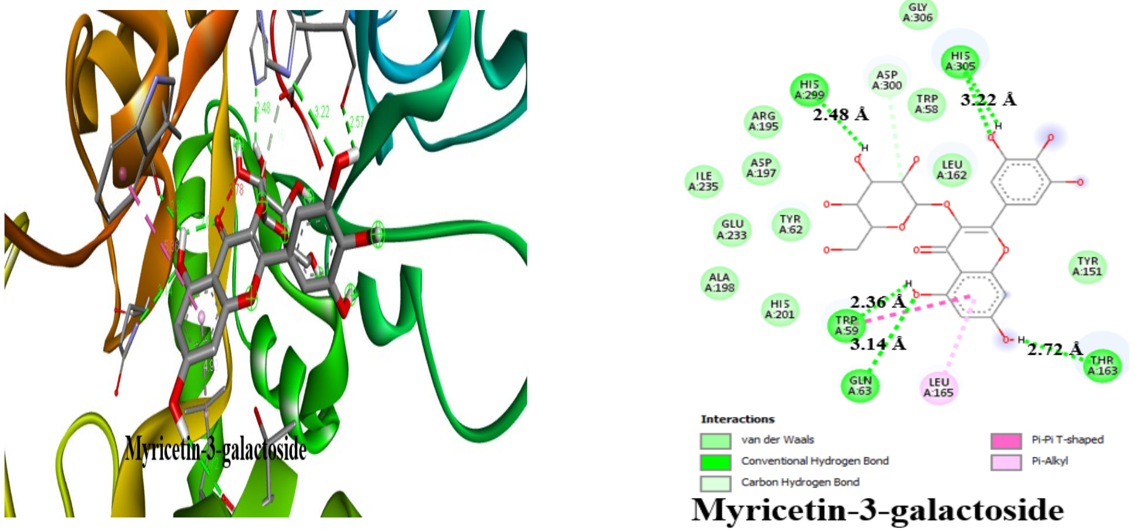
Myricetin-3-galactoside
GLY A:306
HIS A:305
ASP A:300
HIS A:299
TRP A:58
2.48 Å
3.22 Å
ARG A:195
ASP A:197
LEU A:162
ILE A:235
GLU A:233
TYR A:62
TYR A:151
ALA A:198
HIS A:201
2.36 Å
TRP A:59
3.14 Å
2.72 Å
THR A:163
GLN A:63
LEU A:165
Interactions
van der Waals
Conventional Hydrogen Bond
Carbon Hydrogen Bond
Pi-Pi T-shaped
Pi-Alkyl
Myricetin-3-galactoside

l

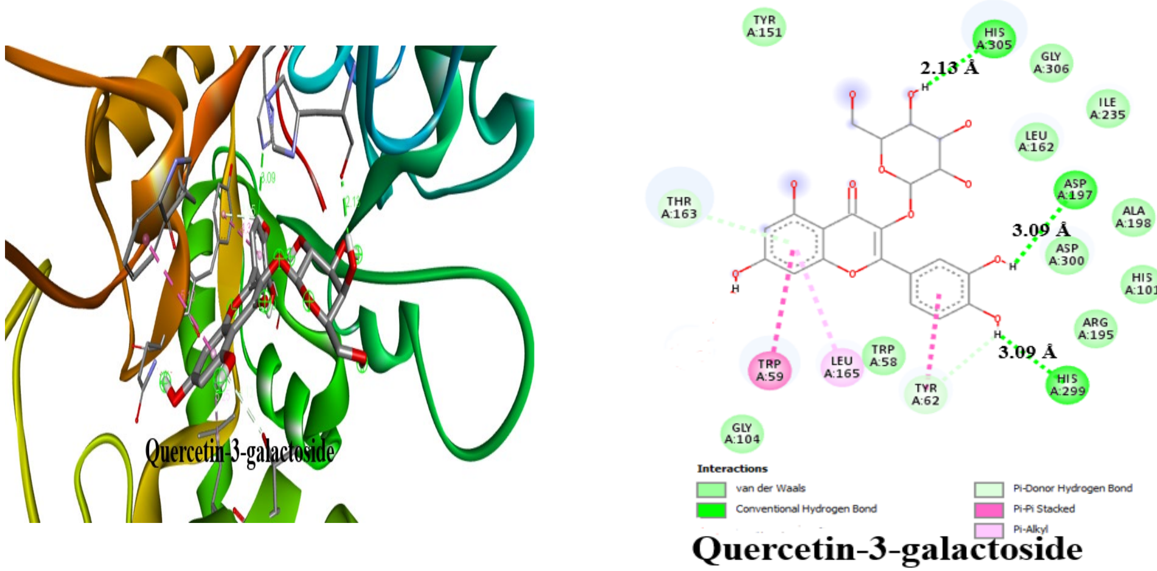
Quercetin-3-galactoside
TYR A:151
HIS A:305
GLY A:306
2.13 Å
ILE A:235
LEU A:162
THR A:163
ASP A:197
ALA A:198
3.09 Å
ASP A:300
HIS A:101
ARG A:195
3.09 Å
TRP A:59
LEU A:165
TRP A:58
TYR A:62
HIS A:299
GLY A:104
Interactions
van der Waals
Conventional Hydrogen Bond
Pi-Donor Hydrogen Bond
Pi-Pi Stacked
Pi-Alkyl
Quercetin-3-galactoside

m

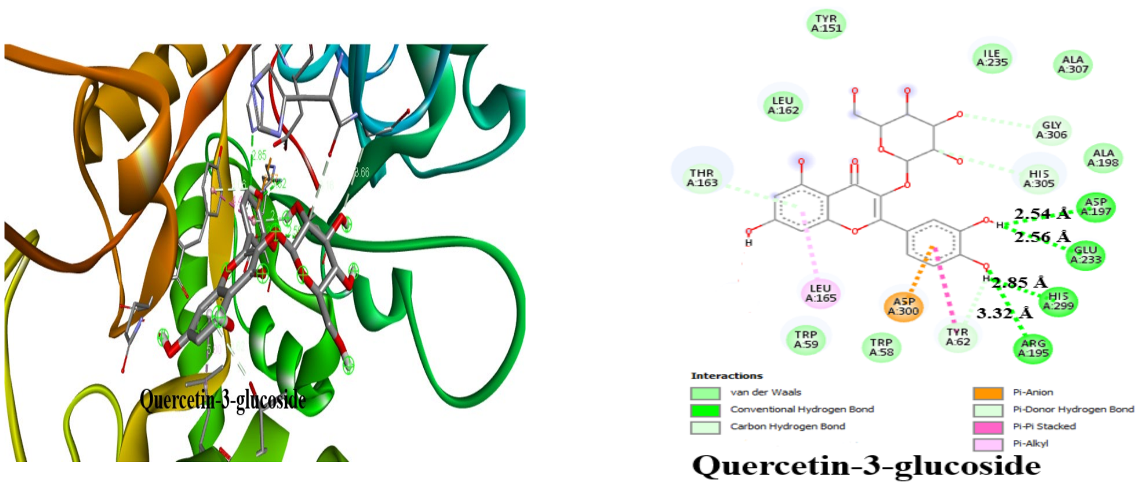
Quercetin-3-glucoside
TYR A:151
ILE A:235
ALA A:307
LEU A:162
GLY A:306
ALA A:198
THR A:163
HIS A:305
ASP A:197
GLU A:233
2.54 Å
2.56 Å
2.85 Å
3.32 Å
LEU A:165
ASP A:300
HIS A:299
TRP A:59
TRP A:58
TYR A:62
ARG A:195
Interactions
van der Waals
Conventional Hydrogen Bond
Carbon Hydrogen Bond
Pi-Anion
Pi-Donor Hydrogen Bond
Pi-Pi Stacked
Pi-Alkyl
Quercetin-3-glucoside

Figure S2. Model of the Interaction and the 2D Structure of α- glucosidase protein with (a) 1,3-Dicaffeoylquinic acid (b) 1,4-Dicaffeoylquinic acid (c) 3,4-Dicaffeoylquinic acid (d) 3,5-Dicaffeoylquinic acid (e) 4,5-Dicaffeoylquinic acid (f) 5-caffeoylquinic acid (g) Cascaroside C (h) Isorhamnetin 3-galactoside (i) Kaempferol galactoside (Trifolin) (j) Methyl 3,5-di-O-caffeoyl quinate (k) Myricetin-3-galactoside (l) Quercetin-3-galactoside (m) Quercetin-3-glucoside.

a

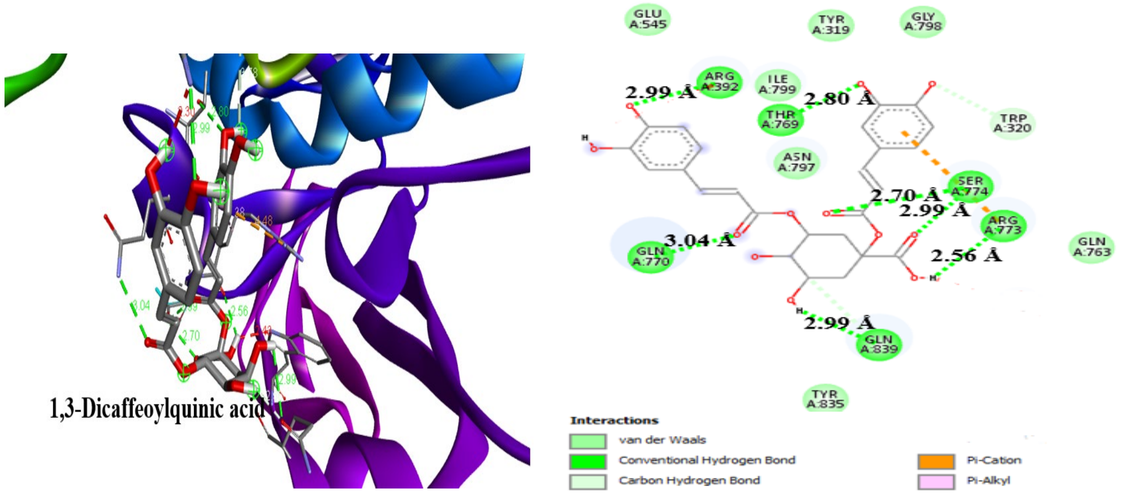

1,3-Dicaffeoylquinic acid

b

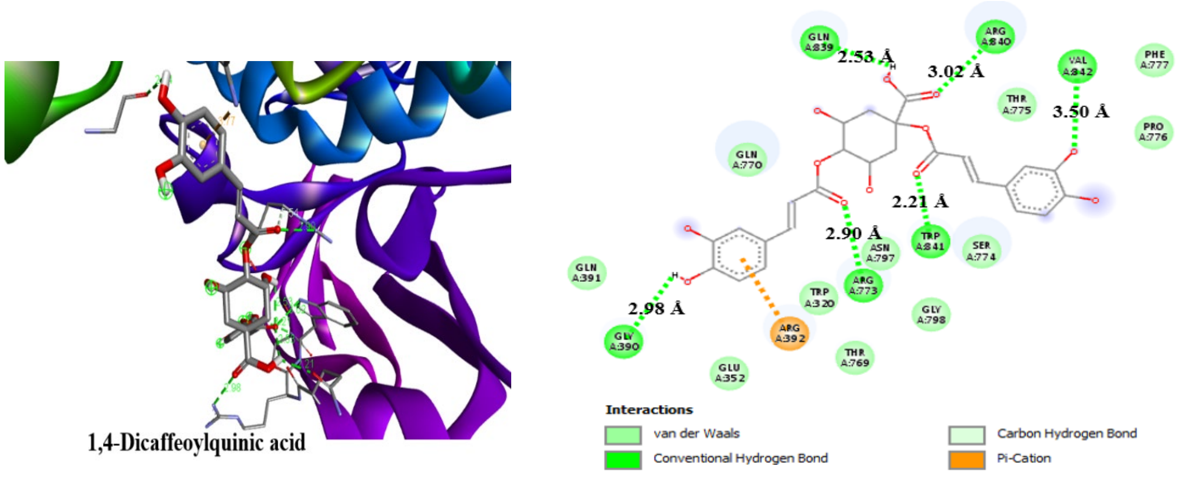

1,4-Dicaffeoylquinic acid

c

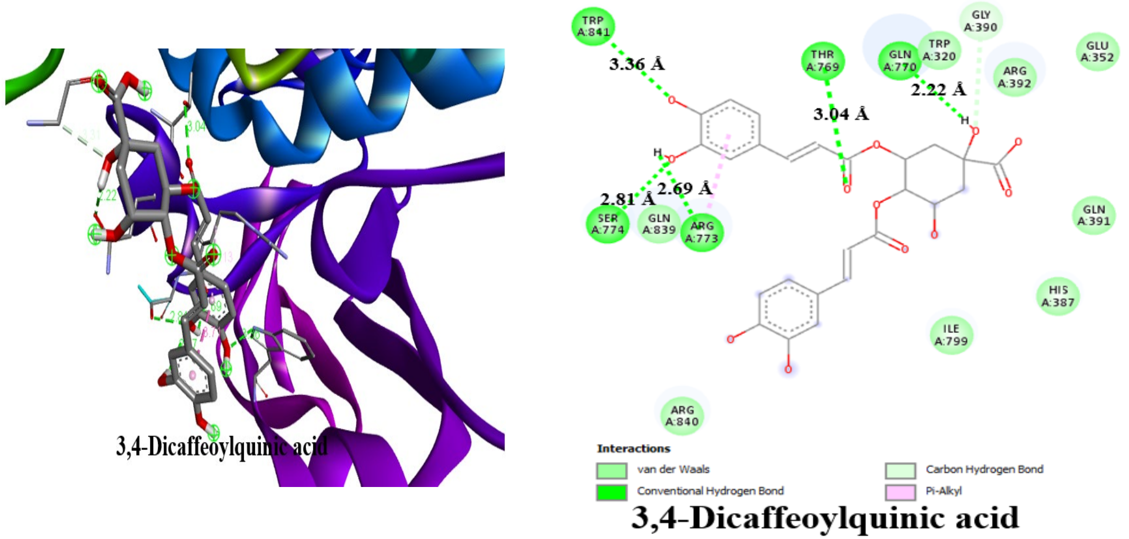
3,4-Dicaffeoylquinic acid
TRP A:841
THR A:769
GLN A:770
TRP A:320
GLY A:390
GLU A:352
ARG A:392
3.36 Å
3.04 Å
2.22 Å
2.81 Å
2.69 Å
SER A:774
GLN A:839
ARG A:773
GLN A:391
HIS A:387
ILE A:799
ARG A:840
Interactions
van der Waals
Conventional Hydrogen Bond
Carbon Hydrogen Bond
Pi-Alkyl
3,4-Dicaffeoylquinic acid

d

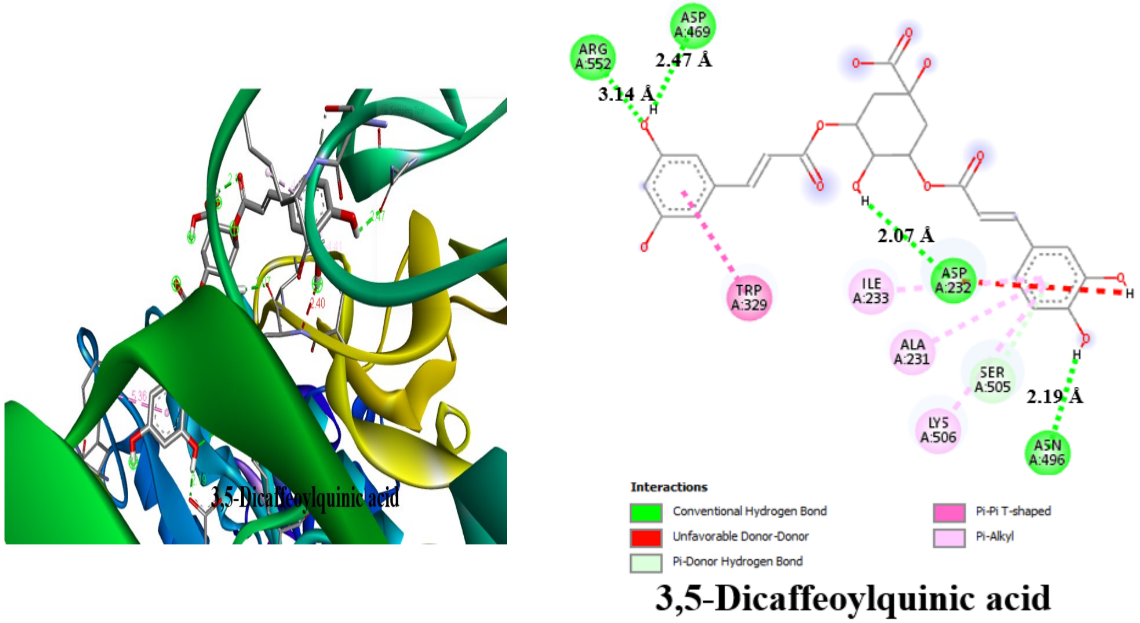
3,5-Dicaffeoylquinic acid
ARG A:552
ASP A:469
2.47 Å
3.14 Å
2.07 Å
TRP A:329
ILE A:233
ASP A:232
ALA A:231
SER A:505
LYS A:506
2.19 Å
ASN A:496
Interactions
Conventional Hydrogen Bond
Unfavorable Donor-Donor
Pi-Donor Hydrogen Bond
Pi-Pi T-shaped
Pi-Alkyl
3,5-Dicaffeoylquinic acid

e

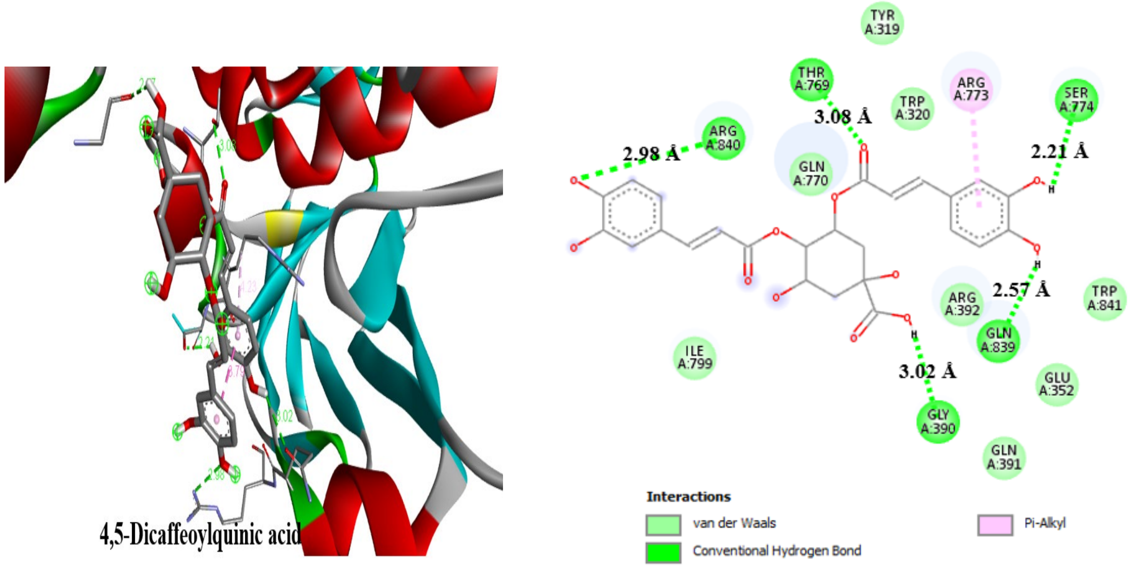

4,5-Dicaffeoylquinic acid

f

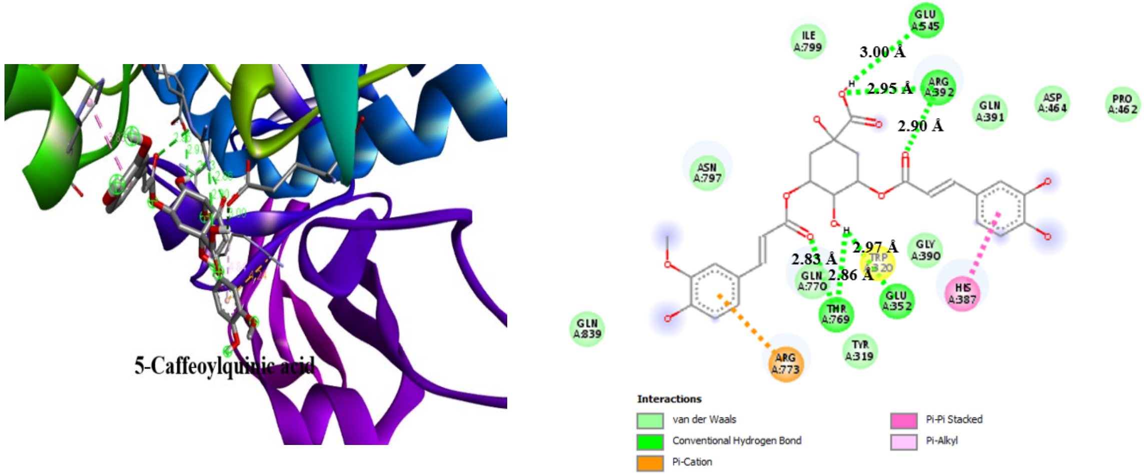

5-Caffeoylquinic acid

g

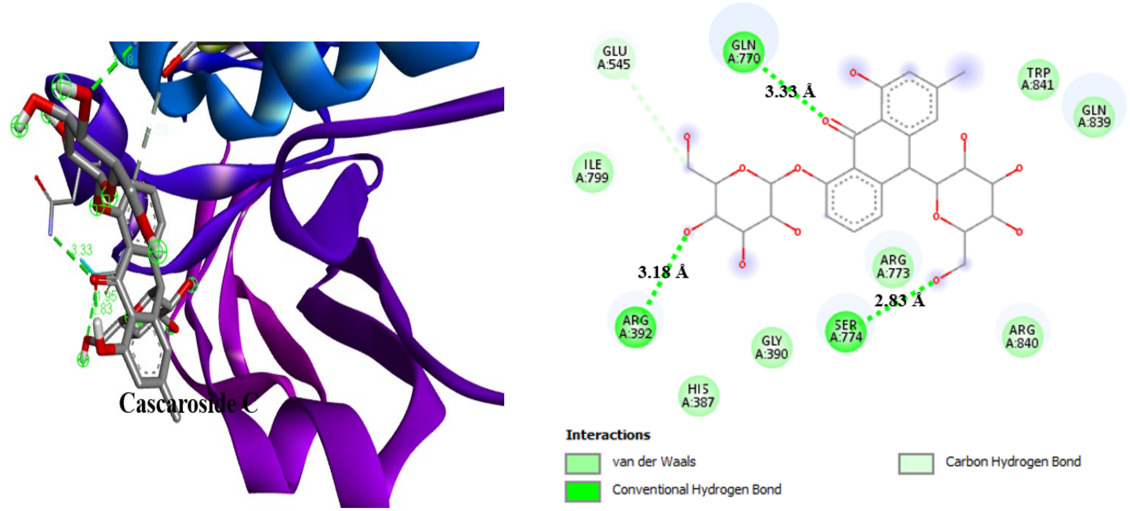

Cascaroside C

h

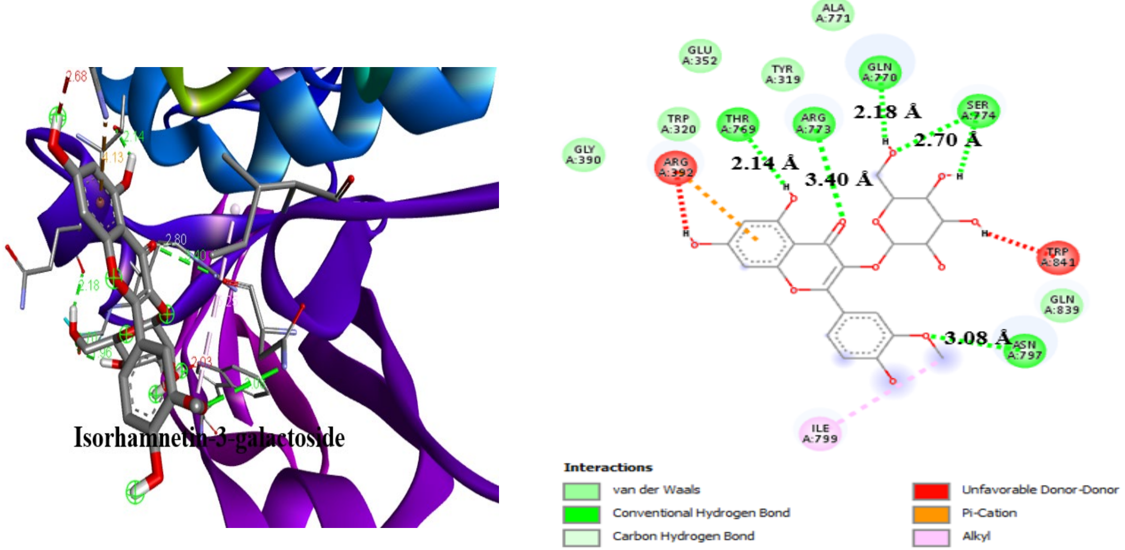

Isorhamnetin-3-galactoside

i

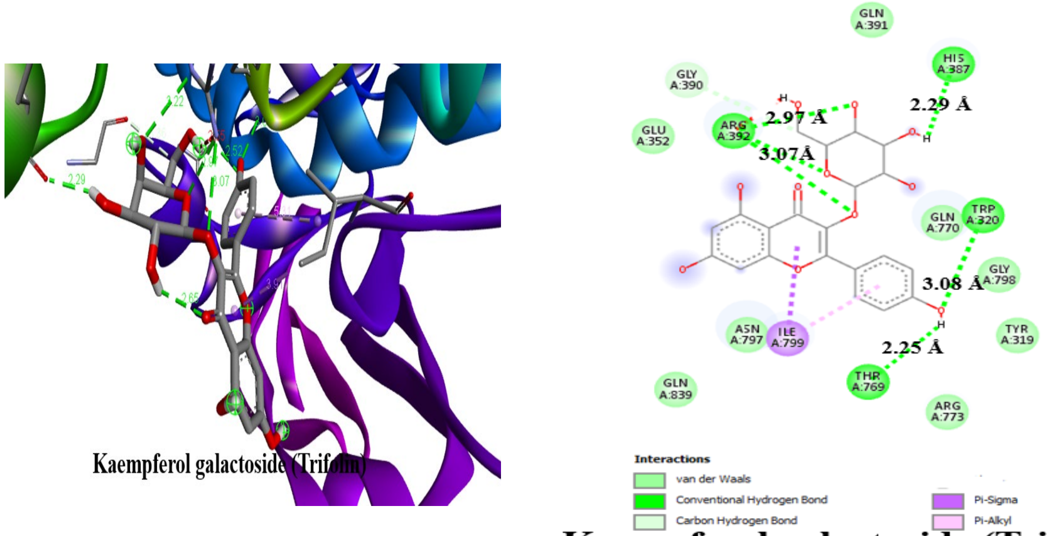

Kaempferol galactoside (Trifolin)

j

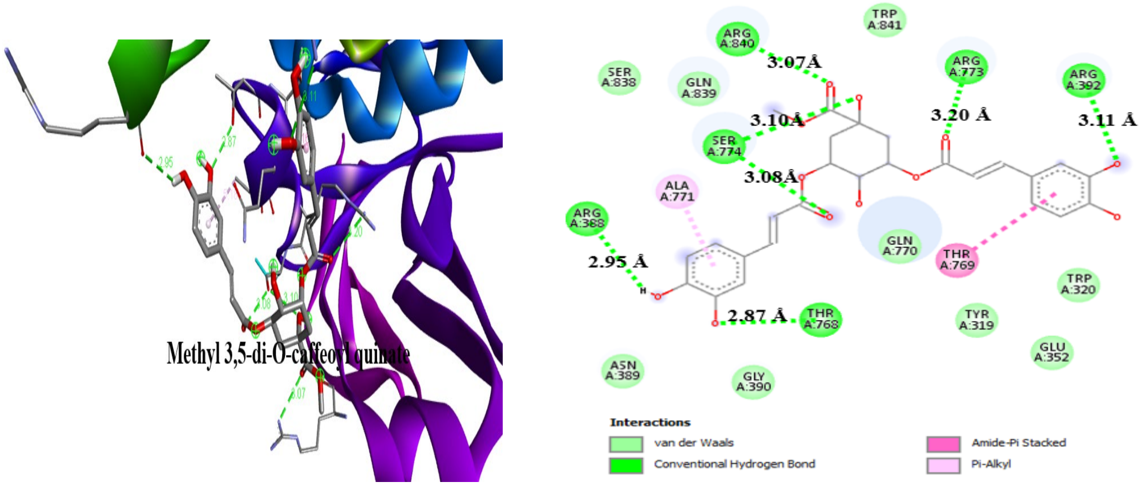

Methyl 3,5-di-O-caffeoyl quinate

k

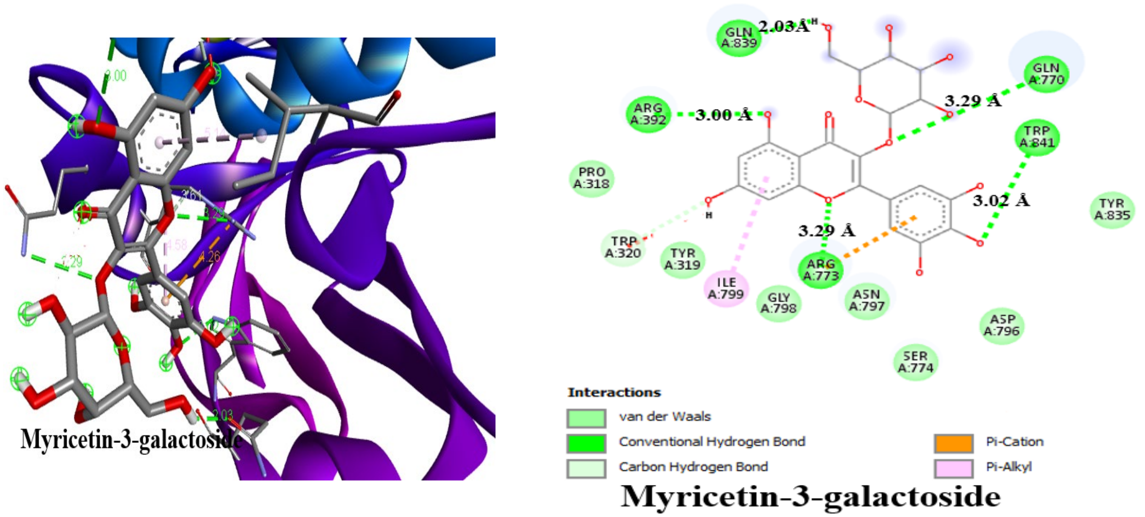
Myricetin-3-galactoside
GLN A:839
2.03 Å
GLN A:770
ARG A:392
3.00 Å
3.29 Å
TRP A:841
PRO A:318
TYR A:835
3.02 Å
TRP A:320
3.29 Å
TYR A:319
ARG A:773
ILE A:799
GLY A:798
ASN A:797
ASP A:796
SER A:774
Interactions
van der Waals
Conventional Hydrogen Bond
Carbon Hydrogen Bond
Pi-Cation
Pi-Alkyl
Myricetin-3-galactoside

l

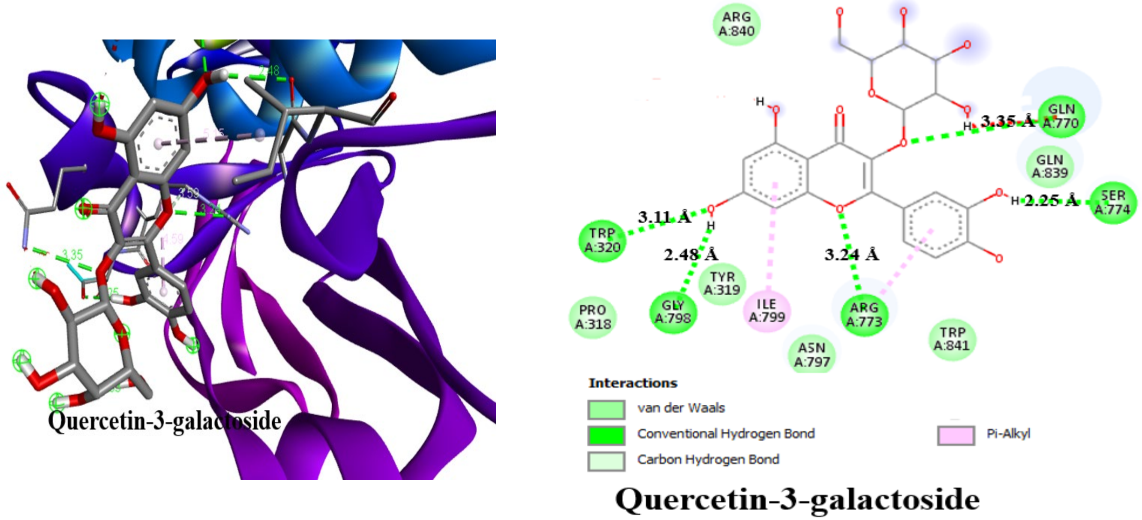
Quercetin-3-galactoside
ARG A:840
3.35 Å
GLN A:770
GLN A:839
2.25 Å
SER A:774
3.11 Å
TRP A:320
2.48 Å
3.24 Å
TYR A:319
PRO A:318
GLY A:798
ILE A:799
ARG A:773
TRP A:841
ASN A:797
Interactions
van der Waals
Conventional Hydrogen Bond
Carbon Hydrogen Bond
Pi-Alkyl
Quercetin-3-galactoside

m

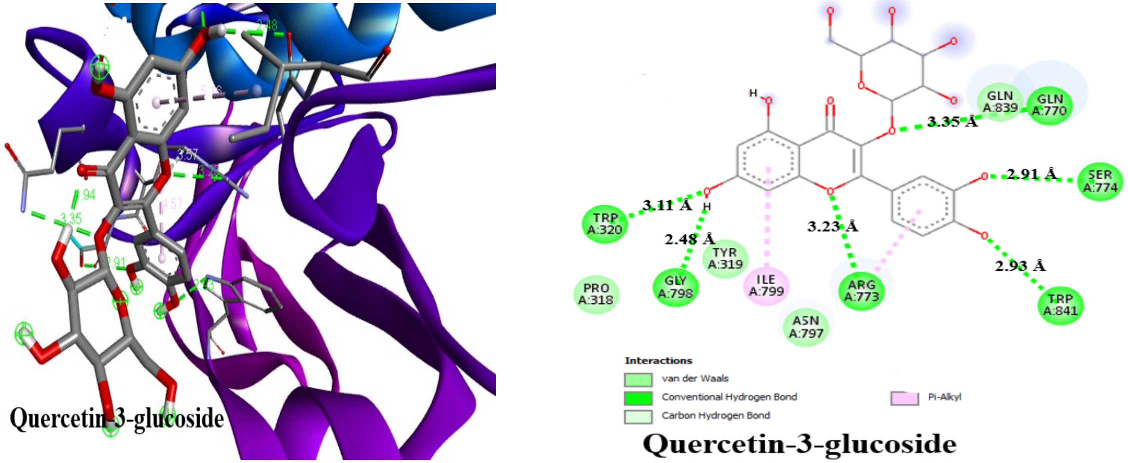
Quercetin-3-glucoside
GLN A:839
GLN A:770
3.35 Å
2.91 Å
SER A:774
3.11 Å
TRP A:320
2.48 Å
3.23 Å
TYR A:319
PRO A:318
GLY A:798
ILE A:799
ARG A:773
2.93 Å
TRP A:841
ASN A:797
Interactions
van der Waals
Conventional Hydrogen Bond
Carbon Hydrogen Bond
Pi-Alkyl
Quercetin-3-glucoside
